# Variational biomarker pooling with calibration for time-to-event outcomes across multiple clinical studies

**DOI:** 10.1186/s12874-026-02827-y

**Published:** 2026-03-23

**Authors:** Jiali Song, Zhiwei Rong, Yan Hou

**Affiliations:** 1 Department of Biostatistics, School of Public Health, Peking University, Beijing, 100083 China; 2 School of Public Health, Tianjin Medical University, Tianjin, 300000 China; 3 Key Laboratory of Carcinogenesis and Translational Research (Ministry of Education), Peking University Cancer Hospital & Institute, Beijing, 100142 China; 4 Peking University Third Hospital Beijing Key Laboratory of Intelligent Neuromodulation and Brain Disorder Treatment, Beijing, 100083 China

**Keywords:** Biomarker pooling, Mean-field variational inference, Inter-study variability, Calibration

## Abstract

**Background:**

Biomarkers are widely used in oncology research to study disease progression and predict survival outcomes. Pooling biomarker data across studies can improve precision, but pooled analyses are often affected by assay heterogeneity. Many studies use calibration designs because re-assaying all biospecimens on a reference platform is impractical. In pooled analyses, calibration can be incomplete when some studies have no reference measurements. This setting can be viewed as covariate measurement error in time-to-event models. Most existing methods were developed for single-cohort designs with validation or replicate measurements, and they do not directly accommodate study-specific calibration with incomplete reference data in pooled survival analyses.

**Methods:**

In this paper, we propose Variational Inference-Based Biomarker Pooling (VIBP) for censored survival data. VIBP treats the target biomarker as latent and jointly models study-specific calibration and the survival outcome using parametric survival models. Variational inference provides scalable estimation, and uncertainty is quantified using bootstrap.

**Results:**

Through extensive simulation studies for exponential and Weibull survival data, we find that VIBP consistently provides estimates with lower bias, smaller mean squared error, and near-nominal 95% coverage across a wide range of effect sizes and censoring rates. We further apply VIBP to a real-world dataset to evaluate the association between DJ-1 protein levels and overall survival in lung squamous cell carcinoma. The results highlight the ability of VIBP to recover meaningful survival associations under sparse and heterogeneous calibration information.

**Conclusions:**

The proposed method provides accurate and robust estimation in the presence of inter-study variability and partially observed reference measurements, and it remains applicable even when some studies have no reference data.

**Supplementary Information:**

The online version contains supplementary material available at 10.1186/s12874-026-02827-y.

## Background

Biomarkers play a central role in medical research and clinical practice, particularly in oncology, where survival and other time-to-event outcomes are often primary endpoints [1, 2]. Quantifying biomarker–survival associations informs prognostic stratification and treatment decision-making [3, 4] and supports the identification of patient subgroups with distinct risk profile [2]. Despite their importance, individual early-phase or translational investigations often lack sufficient sample sizes to reliably detect biomarker-survival associations [3]. To address this limitation, biomarker pooling across multiple studies is widely used to improve statistical power and robustness, and to support more generalizable inference [5]. A key challenge in pooled biomarker analyses is separating true between-study biological differences from measurement heterogeneity introduced by different assays, kits, laboratories, and operational procedures [6].

Biomarker measurements can exhibit substantial inter-assay and inter-laboratory variability. For example, the between-laboratory coefficient of variation often exceeds 25% for estrone and estradiol measurements [7]. Although adopting more stable platforms such as RNA sequencing can reduce measurement heterogeneity [8–11], re-assaying all biospecimens on a new platform is often infeasible. Consequently, calibration designs are commonly used, in which only a subset of samples is re-assayed on the new reference platform to provide *reference measurements*, while the original measurements are treated as *local measurements*. In practice, similar calibration structures also arise when integrating information across different omics layers. From a biological perspective, protein abundance is typically more proximal to disease mechanisms than gene expression. However, protein assays are costly and often available only for a subset of subjects, while gene expression is broadly collected. Treating gene expression as a direct substitute for protein levels may therefore yield attenuated and less interpretable survival associations, motivating methods that explicitly account for an indirectly and noisily observed target biomarker.

Pooled and calibrated biomarker data can be viewed as a covariate measurement error problem for time-to-event outcomes, with reference measurements approximating the latent true biomarker and local measurements serving as surrogates [12–14]. A substantial literature addresses measurement error correction in hazard regression. Regression calibration and risk-set calibration use calibrated predictors that are often derived from validation or reliability information to mitigate bias in hazard regression [15, 16]. SIMEX extrapolates estimates to the zero-error limit by adding artificial noise [17]. Corrected or conditional score methods modify estimating equations to achieve consistency under specified error models [18, 19]. Full-likelihood or joint modeling approaches treat the true covariate as latent and jointly model the measurement and survival processes to propagate uncertainty into inference on survival associations [13].

Despite substantial progress in measurement error correction for time-to-event outcomes, existing methods have primarily been developed for single-cohort designs and are not directly tailored to calibration-based multi-study pooling [13, 14]. To our knowledge, the closest related pooling approach is by Solan et al., but it is limited to binary endpoints [5, 6, 20], leaving survival outcomes largely unaddressed. In pooled translational studies, calibration relationships are often study-specific and calibration designs are frequently incomplete, with some studies lacking reference measurements entirely. Under these conditions, widely used two-stage calibration procedures may rely on simplifying assumptions and typically do not fully propagate calibration uncertainty to survival effect estimation [6]. These limitations can be exacerbated in oncology studies with substantial biomarker effects and censoring, where the hazard model’s exponential link can magnify the impact of biomarker variability and measurement error.

Building on our previous work on biomarker pooling for binary outcomes [21], we propose a new method termed Variational Inference-based Biomarker Pooling (VIBP), a unified framework for calibration-based multi-study pooling with censored time-to-event outcomes. The proposed method treats the true biomarker level as a latent variable and jointly models the calibration process and censored survival outcomes within a unified Bayesian framework. Estimation is performed using mean-field variational inference instead of sampling-based methods to ensure computational efficiency, and uncertainty is quantified using bootstrap-based confidence intervals. The main contributions of this work are as follows: (i) extension of calibration-based pooling to censored time-to-event outcomes; (ii) explicit accommodation of incomplete calibration designs, including study-level structural missingness; (iii) development of a computationally efficient variational algorithm for joint calibration-survival modeling, supported by extensive simulations and a real-data application. The remainder of this article is organized as follows. Sect. "Methods" describes the statistical framework and implementation of the VIBP model. Sect. "Simulation studies" presents simulation studies to demonstrate the efficacy of our approach under various theoretical scenarios. Sect. "Application and case study" applies the proposed method to a real-world dataset to illustrate its practical utility. Sect. "Discussion" discusses the implications, potential limitations, and the broader relevance of our findings.

## Methods

### Mathematical notation and model assumptions

Let $s=1,\dots ,S$ index the studies contributing to the pooling analysis, and let ${n}_{s}$ denote the sample size of study $s$. The total number of individuals from all studies is $N=\sum_{s=1}^{S}{n}_{s}$. For individual $i$ in study $s$, let ${y}_{si}=\left({t}_{si},{\delta }_{si}\right)$ denote the survival outcome, where ${t}_{si}$ is the observed follow-up time and ${\delta }_{si}=1$ if the event is observed and ${\delta }_{si}=0$ otherwise. Let ${x}_{si}$ be the biomarker measurement on the new platform (reference measurement), ${w}_{si}$ the biomarker measurement from the original assay (local measurement), and ${z}_{si}$ a ${p}_{z}$-dimensional vector of covariates. In the motivating application, ${x}_{si}$ can correspond to protein measurements obtained from a reference platform, whereas ${w}_{si}$ represents gene expression measurements from the original assay.

We assume that all individuals have the local measurements ${w}_{si}$, but only a subset have reference measurements ${x}_{si}$. Define ${I}_{si}=1$ if ${x}_{si}$ is observed and ${I}_{si}=0$ otherwise. The reference biomarker measurements ${x}_{si}$ are assumed to follow the same distribution across studies because reference platform is standardized, while the distribution of local measurements ${w}_{si}$ may vary by study due to platform-specific effects. We further assume conditionally independent of the survival outcome ${y}_{si}$ and the local measurement ${w}_{si}$ given the reference measurement ${x}_{si}$ and the covariates ${z}_{si}$, such that

1 $$\begin{array}{c}p\left({y}_{si},{w}_{si}|{x}_{si},{z}_{si}\right)=p\left({w}_{si}|{x}_{si}\right)p\left({y}_{si}|{x}_{si},{z}_{si}\right)\end{array}.$$

    

For simplicity, we further assume that covariates ${z}_{si}$ do not affect the calibration of ${w}_{si}$, that is,

2 $$\begin{array}{c}p\left({w}_{si}|{x}_{si},{z}_{si}\right)=p\left({w}_{si}|{x}_{si}\right)\end{array}.$$

Let $y={\left({y}_{11},\dots ,{y}_{1{n}_{1}},\dots ,{y}_{S1},\dots ,{y}_{S{n}_{S}}\right)}^{T}$, $w={\left({w}_{11},\dots ,{w}_{1{n}_{1}},\dots ,{w}_{S1},\dots ,{w}_{S{n}_{S}}\right)}^{T}$, and $Z={\left({{z}}_{11},\dots ,{{z}}_{1{n}_{1}},\dots ,{{z}}_{S1},\dots ,{{z}}_{S{n}_{S}}\right)}^{T}$. Let ${x}^{o}=\left\{{x}_{si}|{I}_{si}=1\right\}$ denote the observed reference measurements and ${x}^{m}=\left\{{x}_{si}|{I}_{si}=0\right\}$ the unobserved ones, so that $x=\left({x}^{o},{x}^{m}\right)$. Let $\theta$ denote the collection of all model parameters.

Under the conditional independence assumptions in Eq. (1, 2), the complete-data log-likelihood of all observations can then be written as

3 $$\begin{array}{c}\mathrm{log}p\left(y,w,x\vert Z,\theta\right)=\sum\limits_{s,i}\left[\mathrm{log}p\left(y_{si}\vert x_{si},z_{si},\theta\right)+\mathrm{log}p\left(w_{si}\vert x_{si},\theta\right)+\mathrm{log}p\left(x_{si}\vert\theta\right)\right]\end{array}.$$

This decomposition comprises three components, that is, the association between biomarker and the survival outcome $p\left({y}_{si}|{x}_{si},{z}_{si},\theta \right)$, the calibration model linking local and reference measurements $p\left({w}_{si}|{x}_{si},\theta \right)$, and the marginal distribution of the reference measurement $p\left({x}_{si}|\theta \right)$.

The calibration model is specified as a linear relationship between ${x}_{si}$ and ${w}_{si}$, assuming normal errors,

4 $$\begin{array}{c}p\left({w}_{si}|{x}_{si},\theta \right)=p\left({w}_{si}|{x}_{si},{a}_{s},{b}_{s},{\sigma }_{ws}^{2}\right)=N\left({w}_{si}|{a}_{s}+{b}_{s}{x}_{si},{\sigma }_{ws}^{2}\right)\end{array},$$

where ${a}_{s}$ and ${b}_{s}$ are study-specific calibration parameters. The reference biomarker values are modelled as

5 $$\begin{array}{c}p\left({x}_{si}|\theta \right)=p\left({x}_{si}|{\mu }_{x},{\sigma }_{x}^{2}\right)=N\left({x}_{si}|{\mu }_{x},{\sigma }_{x}^{2}\right)\end{array}.$$

The observed-data likelihood $\mathrm{log}p\left(y,w,{x}^{o}|Z,\theta \right)$ is obtained by integrating Eq. (3) over the unobserved ${{x}}^{m}$, which is analytically intractable. In addition, the outcome component $p\left({y}_{si}|{x}_{si},{z}_{si},\theta \right)$ is specified using either an exponential or Weibull survival model, which further complicates direct maximization. Therefore, we adopt a variational inference approach to estimate $\theta$, which provides a stable and computationally efficient approximation to the posterior in the presence of high-dimensional latent variables (the unobserved ${{x}}^{m}$) and repeated model fitting in resampling-based inference.

For exponential survival models, we refer to the resulting estimator as VIBPe. For Weibull survival models, we refer to the resulting estimator as VIBPw, where the variational updates additionally involve the shape parameter $\rho$. To account for uncertainty in $\rho$, we considered two prior specifications within the VIBPw framework. A gamma prior leads to the estimator VIBPwg, and a lognormal prior leads to the estimator VIBPwl. These variants share the same biomarker calibration structure and variational updates for the regression coefficients, but differ in the prior-to-posterior approximation for $\rho$ induced by the chosen prior.

### Variational inference for survival outcomes with an exponential distribution

#### Survival outcome model

To model the association between the biomarker and the survival outcome, we first consider an exponential survival model, which assumes a constant hazard over time. Under this model, the hazard function does not depend on time since study entry. Using the well-known equivalence between the exponential survival likelihood and a Poisson likelihood with event indicator ${\delta }_{si}\in \left\{\mathrm{0,1}\right\}$, we express the outcome model as a Poisson generalized linear model

6 $$\begin{array}{c}{\delta }_{si}\sim \mathrm{Poisson}\left({\mu }_{si}\right),\\{\mu }_{si}={t}_{si}{e}^{{\eta }_{si}},\\{\eta }_{si}={\beta }_{0s}+{\beta }_{x}{x}_{si}+{\beta }_{z}^{\top }{z}_{si},\end{array}$$

where ${\eta }_{si}$ is the linear predictor, ${\beta }_{0s}$ is the study-specific intercept, ${\beta }_{x}$ is the primary parameter of interest, describing the relationship between the biomarker and the outcome, ${\beta }_{z}={\left({\beta }_{z1},\dots ,{\beta }_{z{p}_{z}}\right)}^{T}$ are regression coefficients for the covariates. We collect all outcome model parameters into

7 $$\begin{array}{c}{\theta }_{\beta }={\left({\beta }_{01},\dots ,{\beta }_{0S},{\beta }_{x},{\beta }_{z}^{T}\right)}^{T}\in {R}^{S+1+{p}_{z}}\end{array}.$$

The individual log-likelihood contribution from the outcome model is

8 $$\begin{array}{c}{l}_{si}\left({\eta }_{si}\right)={\delta }_{si}{\eta }_{si}-{t}_{si}{e}^{{\eta }_{si}}\end{array}.$$

To obtain conditionally conjugate variational factors such as Gaussian or Inverse-Gamma, we replace the non-quadratic log-likelihood in Eq. (8) with a local quadratic approximation. Following the iteratively reweighted least squares (IRLS) construction [22, 23], we approximate each term ${l}_{si}\left({\eta }_{si}\right)$ by a second-order expansion around the current variational mean ${m}_{\eta ,si}={E}_{q}\left[{\eta }_{si}\right]$,

9 $$\begin{array}{c}{l}_{si}\left({\eta }_{si}\right)\approx -\frac{1}{2}{{\omega }_{si}\left({\eta }_{si}-{z}_{si}^{*}\right)}^{2}+\mathrm{const},\end{array}$$

where

$$\begin{array}{c}{\omega }_{si}={\overline{\mu }}_{si}={t}_{si}{E}_{q}\left[{e}^{{\eta }_{si}}\right],\\{z}_{si}^{*}={m}_{\eta ,si}+\frac{{\updelta }_{si}-{\overline{\mu }}_{si}}{{\overline{\mu }}_{si}}.\end{array}$$

This IRLS-based quadratic surrogate transforms the exponential likelihood into a conditionally Gaussian form in ${\eta }_{si}$, which in turn yields closed-form variational updates for the regression parameters.

#### Prior distributions

Throughout, we use superscript 0 to denote the hyperparameters. For the outcome model parameters ${\theta }_{\beta }$, we recommend informative or weakly informative priors to maintain power and avoid serious instability. We adopt a multivariate normal prior,

10 $$\begin{array}{c}{\theta }_{\beta }\sim N\left({m}_{\beta }^{0},{\Sigma }_{\beta }^{0}\right),\\{\Sigma }_{\beta }^{0}=\mathrm{diag}\left({\sigma }_{0}^{2}{I}_{s},{\Sigma }_{glob}^{0}\right),\end{array}$$

where ${\sigma }_{0}^{2}$ is the prior variance of the study-specific intercepts $\left({\beta }_{01},\dots ,{\beta }_{0S}\right)$, ${I}_{s}$ is the $S\times S$ identity matrix, and ${\Sigma }_{glob}^{0}$ is the prior covariance matrix for $\left({\beta }_{x},{\beta }_{z}^{T}\right)$.

For study-specific parameters ${a}_{s}$, ${b}_{s}$, ${\sigma }_{ws}^{2}$, we introduce ${u}_{s}={\left({a}_{s},{b}_{s}\right)}^{T}.$ We assume that ${\left\{{u}_{s},{\sigma }_{ws}^{2}\right\}}_{s=1}^{S}$ are independent draws from a common population distribution, corresponding to an exchangeable random-effects specification,

11 $$\begin{array}{c}{u}_{s}\sim N\left({m}_{ab}^{0},{\Sigma }_{ab}^{0}\right),\end{array}$$

and

12 $$\begin{array}{c}{\sigma }_{ws}^{2}\sim \mathrm{InvGamma}\left({\alpha }_{w,}^{o}{\gamma }_{w}^{0}\right)\end{array}.$$

For the marginal distribution of the biomarker ${x}_{si}$, we assign a Normal–Inverse-Gamma prior to $\left({\mu }_{x},{\sigma }_{x}^{2}\right)$, the conjugate family for a normal likelihood with unknown mean and variance. This choice yields closed-form variational updates, since the complete conditional remains Normal–Inverse-Gamma under the mean-field factorization,

13 $$\begin{array}{c}{\sigma }_{x}^{2}\sim \mathrm{InvGamma}{\left({\alpha }_{x,}^{0}{\gamma }_{x}^{0}\right)}_{,}\\{\mu }_{x}|{\sigma }_{x}^{2}\sim N\left({m}_{x,}^{0}\frac{{\sigma }_{x}^{2}}{{\kappa }_{x}^{0}}\right).\end{array}$$

#### Mean-field variational inference via coordinate ascent

We use a mean-field variational approximation and perform coordinate ascent variational inference (CAVI) to maximize the evidence lower bound (ELBO) [24]. It factorizes the joint variational distribution over model parameters and latent variables as

14 $$\begin{array}{c}q\left(\theta ,x\right)=q\left({\theta }_{\beta }\right)\prod\limits_{s=1}^{S}q\left({u}_{s}\right)q\left({\sigma }_{ws}^{2}\right)\prod\limits_{(s,i):{I}_{si}=0}q\left({x}_{si}\right)q\left({\mu }_{x},{\sigma }_{x}^{2}\right),\end{array}$$

where $\theta$ denotes the collection of all unknown parameters.

The ELBO is expressed as

15 $${\mathcal{L}}\left(q\right)= E_q\left[\mathrm{log}\;p\left(y, w,x, \theta|Z\right)\right]-E_{q}\left[\mathrm{log}{q}\left(x,\theta\right)\right].$$

Each variational factor is obtained by the standard coordinate ascent update

16 $$\begin{array}{c}\mathrm{log}{q}^{*}\left({\theta }_{j}\right)={E}_{q\left({\theta }_{-j,x}\right)}\left[\mathrm{log}p\left(y,w,x,\theta \right)\right]+\mathrm{const},\end{array}$$

for ${\theta }_{j}$ denoting any block of parameters (or latent variables) and ${\theta }_{-j}$ all remaining blocks. The detailed derivations of the variational updates are given in Supplementary Section S.A. Here we summarize the resulting closed-form updates.

##### Variational posterior for the outcome model parameters ${\theta }_{\beta }$

The variational posterior for $\theta_\beta$ is multivariate normal,

17 $$\begin{array}{c}q\left({\theta }_{\beta }\right)=N\left({m}_{\beta },{\Sigma }_{\beta }\right),\end{array}$$

with

$$\begin{array}{c}\textstyle\Sigma_\beta=\left(\textstyle\underset{s,i}\sum{\omega}_{s,i}\;E_{q}\left[\psi_{si}\psi{^T_{si}}\right]+\left(\Sigma{^0_\beta}\right)^{-1}\right)^{-1},\\m_{\beta}={\Sigma}{_\beta}\left(\textstyle\underset{s,i}\sum{\omega}_{s,i}Z{^*_{si}}E_{q}\left[\psi_{si}\right]+\left(\Sigma{^0_{\beta}}\right)^{-1}m{^0_{\beta}}\right),\end{array}$$

where the ${\psi }_{si}$ is the design vector consisting of the study-specific intercept (with a ‘1’ in the position corresponding to study $s$), the covariates ${z}_{si}$, and the biomarker term (using ${x}_{si}$ when observed and ${E}_{q}\left[{x}_{si}\right]$ otherwise).

##### Variational posterior of the study-specific parameters $\left({u}_{s},{\sigma }_{ws}^{2}\right)$

For each study $s$ ,  

18 $$\begin{array}{c}q\left({u}_{s}\right)=N\left({m}_{ab,s},{\Sigma }_{ab,s}\right),\end{array}$$

with

$${\Sigma }_{ab,s}={\left({E}_{q}\left[{\sigma }_{ws}^{-2}\right]\sum_{i}{E}_{q}\left[{\Phi }_{si}{\Phi }_{si}^{T}\right]+{\left({\Sigma }_{ab}^{0}\right)}^{-1}\right)}^{-1},$$

$${m}_{ab,s}={\Sigma }_{ab,s}\left({E}_{q}\left[{\sigma }_{ws}^{-2}\right]\sum_{i}{w}_{si}{E}_{q}\left[{\Phi }_{si}\right]+{\left({\Sigma }_{ab}^{0}\right)}^{-1}{m}_{ab}^{0}\right),$$

where ${\Phi }_{si}={\left(1,{x}_{si}\right)}^{T}$, with ${x}_{si}$ replaced by ${E}_{q}\left[{x}_{si}\right]$ when missing.

The variational posterior for ${\sigma }_{ws}^{2}$ is Inverse-Gamma:

19 $$\begin{array}{c}q\left({\sigma }_{ws}^{2}\right)=\mathrm{InvGamma}\left({\alpha }_{w,s},{\gamma }_{w,s}\right)\end{array},$$

with

$$\begin{array}{c}\alpha_{w,s} = \frac{n_{s}}{2} + \alpha^{0}_{w}, \\\gamma_{w,s} = \frac{1}{2} \sum\limits^{n_{s}}_{i=1} E_{q} [(w_{si} - u^{T}_{s} \phi_{si})^{2}] + \gamma^{0}_{w}.\end{array}$$

    

##### Variational posterior for missing reference measurements $x_{si}$ 

For subjects with ${I}_{si}=0$,

20 $$\begin{array}{c}q\left({x}_{si}\right)=N\left({m}_{x,si},{v}_{x,si}\right),\end{array}$$

where

$${v}_{x,si}={\left({\omega }_{si}{E}_{q}\left[{\beta }_{x}^{2}\right]+{E}_{q}\left[{\sigma }_{ws}^{-2}\right]{E}_{q}\left[{b}_{s}^{2}\right]+{E}_{q}\left[{\sigma }_{x}^{-2}\right]\right)}^{-1},$$

$${m}_{m,si}={v}_{m,si}\left(\begin{array}{c}{\omega }_{si}{E}_{q}\left[{\beta }_{x}\right]\left({z}_{si}^{*}-{E}_{q}\left[{\beta }_{0s}\right]-{E}_{q}\left[{\beta }_{z}^{T}{z}_{si}\right]\right)\\ + {E}_{q}\left[{\sigma }_{ws}^{-2}\right]{E}_{q}\left[{b}_{s}\right]\left({w}_{si}-{E}_{q}\left[{a}_{s}\right]\right)+{E}_{q}\left[{\sigma }_{x}^{-2}\right]{E}_{q}\left[{\mu }_{x}\right]\end{array}\right).$$

##### Variational posterior $\left(\mu_{x},\sigma{^2_{x}}\right)$ 

Define the variational sufficient statistics,

21 $$\begin{array}{c}\overline{x }=\frac{1}{N}\sum_{s,i}{E}_{q}\left[{x}_{si}\right], \\{S}_{{x}^{2}}=\sum_{s,i}{E}_{q}\left[{x}_{si}^{2}\right].\end{array}$$

The Normal–Inverse-Gamma update are

22 $$\begin{array}{c}q\left({\sigma }_{x}^{2}\right)=\mathrm{InvGamma}\left({\alpha }_{x}{\gamma }_{x}\right)\end{array},$$

23 $$\begin{array}{c}q\left({\mu }_{x}|{\sigma }_{x}^{2}\right)=N\left({{m}_{x}}_{,}\frac{{\sigma }_{x}^{2}}{{\kappa }_{x}}\right)\end{array},$$

with

$$\begin{array}{c} \alpha_{x} = \frac{N}{2} + \alpha^{0}_{x}, \\\gamma_{x} = \frac{1}{2} \left[(S_{x^{2}} - N\bar{x}^2) +\frac{N \kappa^{0}_{x}(\bar{x} - m^{0}_{x})^{2}}{N + \kappa^{0}_{x}}\right] + \gamma^{0}_{x},\end{array}$$

$$\begin{array}{c}{m}_{x}={\frac{N\overline{x }+{\kappa }_{x}^{0}{m}_{x}^{0}}{N+{\kappa }_{x}^{0}}}, \\{\kappa }_{x}=N+{\kappa }_{x}^{0}.\end{array}$$

**Figure:**
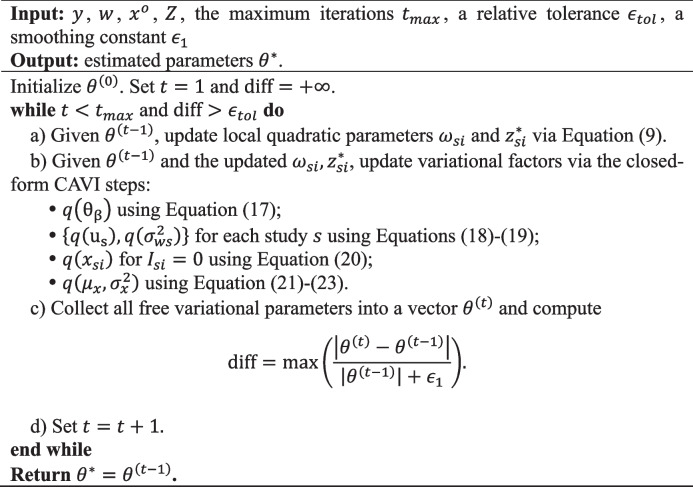
**Algorithm 1** Variational inference for biomarker pooling with exponential survival outcomes (VIBPe)

### Variational inference for survival outcomes with Weibull distribution

In many applications, the assumption of a constant hazard, or equivalently exponentially distributed survival times, may be unrealistic. We therefore extend the outcome model in Sect. "Variational inference for survival outcomes with an exponential distribution" to a Weibull proportional hazards model with shape parameter $\rho>0$. This extension leads to two Weibull-based estimators, that is, VIBPwg under a gamma prior for $\rho$, and VIBPwl under a lognormal prior.

#### Survival outcome model

Under a Weibull proportional hazards model, we adopt the proportional hazards parameterization in which the baseline cumulative hazard is $H\left(t\right)={t}^{\rho }$. Because the scale parameter is not separately identifiable from the study-specific intercepts, we fix the scale to 1 and allow the intercept ${\beta }_{0s}$ to capture the baseline hazard for study $s$. The hazard function is

24 $$\begin{array}{c}h\left({t}_{si}|{\eta }_{si}\right)=\rho {t}_{si}^{\rho -1}{e}^{{\eta }_{si}},\end{array}$$

where the linear predictor ${\eta }_{si}$ is defined as in Sect. "Survival outcome model". The individual log-likelihood contribution is

25 $$\begin{array}{c}{l}_{si}\left({\eta }_{si};\rho \right)={\delta }_{si}\left[\mathrm{log}\rho +(\rho -1)\mathrm{log}{t}_{si}+{\eta }_{si}\right]-{t}_{si}^{\rho }{e}^{{\eta }_{si}}\end{array}$$

Equation (25) is also non-quadratic in ${\eta }_{si}$ as in the exponential case. To obtain conditionally conjugate variational factors, we again adopt an IRLS-based local quadratic approximation [22, 23]. Specifically, at each variational iteration, the log-likelihood is replaced by a second-order Taylor expansion around the current variational mean ${m}_{\eta ,si}=E\left[{\eta }_{si}\right]$.

26 $$\begin{array}{c}{l}_{si}\left({\eta }_{si};\rho \right)\approx -\frac{1}{2}{{\omega }_{si}\left({\eta }_{si}-{z}_{si}^{*}\right)}^{2}+\mathrm{const},\end{array}$$

where

$$\begin{array}{c}{\omega }_{si}={\overline{\mu }}_{si}={{t}_{si}}^{\rho }E\left({e}^{{\eta }_{si}}\right), \\{z}_{si}^{*}={m}_{\eta ,si}+\frac{{\updelta }_{si}-{\overline{\mu }}_{si}}{{\overline{\mu }}_{si}}.\end{array}$$

This surrogate has the same quadratic form as in the exponential case, but with ${\omega }_{si}$ scaled by the Weibull factor ${{t}_{si}}^{\rho }$. The resulting Gaussian representation of ${\eta }_{si}$ permits closed-form variational updates within the Weibull-based VIBP algorithm. The exponential survival model is recovered as a special case when $\rho =1$.

#### Prior distributions for the shape parameter

For the Weibull shape parameter $\rho$, we consider two prior specifications. First, we use a Gamma prior,

27 $$\begin{array}{c}\rho \sim \mathrm{Gamma}\left({\alpha }_{\rho }^{0},{\upgamma }_{\rho }^{0}\right),\end{array}$$

which restricts $\rho$ to be positive and provides a flexible one-dimensional prior. Second, we consider a lognormal prior by introducing $\upxi =\mathrm{log}\rho$ and setting

28 $$\begin{array}{c}\xi \sim N\left({m}_{\upxi }^{0},{\left({\sigma }_{\upxi }^{2}\right)}^{0}\right),\end{array}$$

so that $\rho ={e}^{\upxi }$. The lognormal prior offers a simple positive parameterization for $\rho$ and allows right-skewed uncertainty. Considering both priors enable us to balance analytic numerical stability and convenience (Gamma prior) and flexibility for representing uncertainty (log-normal prior) in applications.

#### Mean-field variational inference with coordinate ascent and updates for ${{\rho}}$

In principle, a fully probabilistic variational treatment could be obtained by specifying a variational distribution $q(\rho )$, for example within the gamma family. We initially explored such a variational approximation (provided in Supplementary Section S.B, which summarizes the Gamma variational specification for $q(\rho )$ and the corresponding updates). However, in practice, the resulting updates were numerically unstable and exhibited slow or erratic convergence in finite samples. One possible explanation is that the Weibull shape parameter enters the likelihood through highly nonlinear functions, which could limit the accuracy of mean-field approximations for $q(\rho )$. We therefore adopt a degenerate variational approximation for $\rho$, in which $q(\rho )$ is set to a Dirac delta distribution at the maximizer of its conditional ELBO component given the other variational factors. Equivalently, $\rho$ is treated as a point-estimated parameter obtained by maximizing the ELBO with respect to $\rho$, while retaining mean-field variational inference for all remaining parameters and latent variables. This strategy can be viewed as a profiled variational optimization and substantially improves numerical stability.

Accordingly, we extend the mean-field approximation in Eq. (14) to include the shape parameter $\rho$,

29 $$\begin{array}{c}q\left(\theta ,\mathrm{x},\rho \right)=q\left(\rho \right)q\left({\theta }_{\beta }\right)\prod\limits_{s=1}^{S}q\left({u}_{s}\right)q\left({\sigma }_{ws}^{2}\right)\prod\limits_{(s,i):{I}_{si}=0}q\left({x}_{si}\right)q\left({\mu }_{x},{\sigma }_{x}^{2}\right),\end{array}$$

where $q\left(\rho \right)$ is degenerate at its maximizer. The variational factors $q\left({\theta }_{\beta }\right)$, $\left\{q\left({u}_{s}\right),q\left({\sigma }_{ws}^{2}\right)\right\}$, $q\left({x}_{si}\right)$, and $q\left({\mu }_{x},{\sigma }_{x}^{2}\right)$ retain the same functional forms and closed-form updates as in Eqs. (17, 18, 19, 20, 21, 22, 23), with ${\omega }_{si}$ and ${z}_{si}^{*}$ now defined in Eq. (26).

##### Gamma prior (VIBPwg)

Under the gamma prior in Eq. (27), the ELBO contribution in $\rho$ (up to an additive constant) can be written as

30 $$\begin{array}{c}J\left(\uprho \right)=\sum\limits_{s,i}\left\{{\delta }_{si}\left[\mathrm{log}\rho +(\rho -1)\mathrm{log}{t}_{si}\right]-{{t}_{si}}^{\rho }{E}_{q}({e}^{{\eta }_{si}})\right\}+\left(\hspace{0.17em}{\alpha }_{\rho }^{0}-1\right)\mathrm{log}\rho -{\upgamma }_{\rho }^{0}\rho \end{array}.$$

Its first and second derivatives are

31 $$\begin{array}{c}g\left(\rho \right)={J}^{\prime}\left(\uprho \right)=\sum\limits_{s,i}\left[\frac{{\delta }_{si}}{\rho }+{\delta }_{si}\mathrm{log}{t}_{si}-{{t}_{si}}^{\rho }\mathrm{log}{t}_{si}{E}_{q}\left({e}^{{\eta }_{si}}\right)\right]+\frac{{\alpha }_{\rho }^{0}-1}{\rho }-{\upgamma }_{\rho }^{0},\end{array}$$

32 $$\begin{array}{c}{g}^{\prime}\left(\rho \right)={J}^{{\prime}{\prime}}\left(\uprho \right)=\sum\limits_{s,i}\left[-\frac{{\delta }_{si}}{{\rho }^{2}}-{{t}_{si}}^{\rho }({\mathrm{log}{t}_{si})}^{2}{E}_{q}({e}^{{\eta }_{si}})\right]-\frac{{\alpha }_{\rho }^{0}-1}{{\rho }^{2}}\end{array}.$$

We perform a Newton–Raphson update for $\rho$ at each outer iteration,

33 $$\begin{array}{c}{\rho }^{t}={\rho }^{t-1}-\frac{g\left({\rho }^{t-1}\right)}{{g}^{\prime}\left({\rho }^{t-1}\right)}\end{array}.$$

##### Lognormal prior (VIBPwl)

Under the lognormal prior in Eq. (28), we work with $\upxi =\mathrm{log}\rho$. The corresponding ELBO contribution is,

34 $$\begin{array}{c}J\left(\upxi \right)=\sum\limits_{s,i}\left\{{\delta }_{si}\left[\upxi +({e}^{\upxi }-1)\mathrm{log}{t}_{si}\right]-{{t}_{si}}^{{e}^{\upxi }}{E}_{q}\left({e}^{{\eta }_{si}}\right)\right\}-\frac{{\left(\upxi -{m}_{\upxi }^{0}\right)}^{2}}{2{\left({\sigma }_{\upxi }^{2}\right)}^{0}}\end{array}.$$

with gradient and Hessian

35 $$\begin{array}{c}g\left(\upxi \right)={J}^{\prime}\left(\upxi \right)=\sum\limits_{s,i}\left\{{\delta }_{si}\left(1+{e}^{\upxi }\mathrm{log}{t}_{si}\right)-{e}^{\upxi }{{t}_{si}}^{{e}^{\upxi }}\mathrm{log}{t}_{si}{E}_{q}\left({e}^{{\eta }_{si}}\right)\right\}-\frac{\upxi -{m}_{\upxi }^{0}}{{\left({\sigma }_{\upxi }^{2}\right)}^{0}},\end{array}$$

36 $${g}^{\prime}\left(\upxi \right)={J}^{{\prime}{\prime}}\left(\upxi \right)=\sum\limits_{s,i}\left\{{\delta }_{si}{e}^{\upxi }\mathrm{log}{t}_{si}-{e}^{\upxi }{{t}_{si}}^{{e}^{\upxi }}\mathrm{log}{t}_{si}\left(1+{e}^{\upxi }\mathrm{log}{t}_{si}\right){E}_{q}\left({e}^{{\eta }_{si}}\right)\right\}-\frac{1}{{\left({\sigma }_{\upxi }^{2}\right)}^{0}}.$$

We update $\upxi$ at each outer iteration,

37 $$\begin{array}{c}{\upxi }^{t}={\upxi }^{t-1}-\frac{g\left({\upxi }^{t-1}\right)}{{g}^{\prime}\left({\upxi }^{t-1}\right)},{\rho }^{t}={e}^{{\upxi }^{t}}\end{array}.$$

**Figure:**
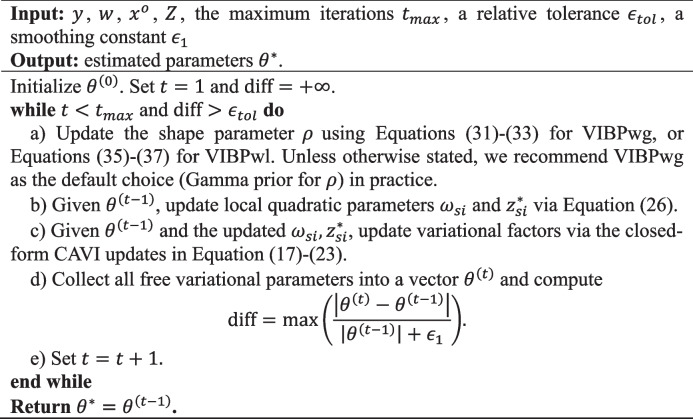
**Algorithm 2** Variational inference for biomarker pooling with Weibull survival outcomes (VIBPw)

### Confidence interval

In empirical analyses, mean-field variational inference often underestimates posterior uncertainty, yielding variational variances that are smaller than those under the exact posterior [24, 25]. To mitigate this limitation, we use the nonparametric bootstrap to construct confidence intervals for the parameters estimated by VIBP, as shown in Algorithm 3 [26]. Default implementation settings (maximum iterations, relative tolerance, smoothing constant, and initialization strategy) used in both simulations and the real-data analysis are provided in Supplementary Section S.C.

**Figure:**
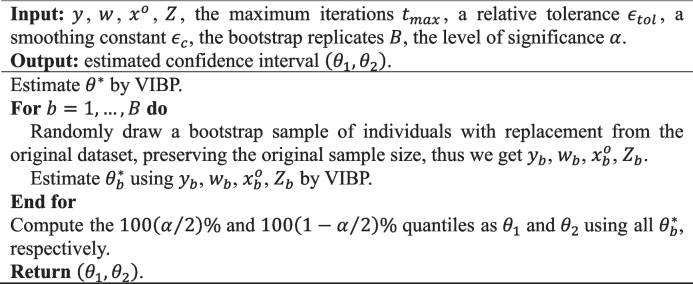
**Algorithm 3** Confidence interval estimation of VIBP

### Compared methods

We compared the proposed VIBP approach with three commonly used methods for estimating biomarker effects from multiple studies, that is naive (${\widehat{\beta }}_{N}$), x-only (${\widehat{\beta }}_{xonly}$), and two-stage (${\widehat{\beta }}_{TS}$) approaches [6, 27].

#### Naive method

The naive method fits a Cox proportional hazards regression model using only the local biomarker measurements, without calibration. The regression coefficient for the biomarker from this model is taken as the effect estimate ${\widehat{\beta }}_{N}$.

#### X-only method

The x-only method fits a Cox proportional hazards regression model using only the observed reference measurements. The analysis is restricted to subjects with reference measurements, and the resulting coefficient for the biomarker is reported as ${\widehat{\beta }}_{xonly}$.

#### Two-stage method

The two-stage method first performs study-specific analyses that adjust for measurement error using regression calibration, and then combines the adjusted estimates in a second stage [27]. Specifically, in the first step, we use regression calibration method to obtain the adjusted effect estimate ${\widehat{\beta }}_{x, s}$ from study $s$. This step can be written as

38 $$\begin{array}{c}{\widehat{\beta }}_{x, s}=\frac{{\widehat{\beta }}_{w, s}}{{\widehat{b}}_{s}},\\\widehat{Var}\left({\widehat{\beta }}_{x, s}\right)={\widehat{b}}_{s}^{-2}\widehat{Var}\left({\widehat{\beta }}_{w, s}\right)+{\widehat{b}}_{s}^{-4}{\widehat{\beta }}_{w, s}^{2}\widehat{Var}\left({\widehat{b}}_{s}\right),\end{array}$$

where ${\widehat{b}}_{s}$ is the estimated calibration slope obtained from the calibration study, ${\widehat{\beta }}_{w, s}$ and $\widehat{Var}({\widehat{\beta }}_{w, s})$ are the naive estimates from a Cox proportional hazards regression model fitted in study $s$ using the local biomarker measurements for individuals not included in the calibration study. In the second step, we combine the study-specific estimates using a fixed-effects meta-analysis. The inverse-variance weights based on $\widehat{Var}\left({\widehat{\beta }}_{x, s}\right)$ are used to obtain a pooled estimate, which are reported as ${\widehat{\beta }}_{TS}$.

## Simulation studies

### Simulation settings

#### Generation of the local and reference laboratory measurements

We consider local and reference laboratory measurements, ${w}_{si}$ and ${x}_{si}$ for individual $i$ in study $s$. Let ${e}_{w,si}$ be the calibration error term in the linear regression of ${w}_{si}$ on ${x}_{si}$. We generate $\left({x}_{si},{w}_{si},{e}_{w,si}\right)$ from a joint multivariate normal distribution for $i=1,\dots ,{n}_{s}, s=1,\dots ,S$,

39 $$\begin{array}{c}\begin{pmatrix}x_{si}\\w_{si}\\e_{w,si}\end{pmatrix}\sim MVN\left(\begin{pmatrix}\mu_x\\a_s+b_s\mu_x\\0\end{pmatrix},\begin{pmatrix}\sigma_x^2&b_s\sigma_x^2&0\\.&b_s^2\sigma_x^2+\sigma_{ws}^2&\sigma_{ws}^2\\.&.&\sigma_{ws}^2\end{pmatrix}\right)\end{array}$$

Equivalently, the data are generated as follows,

40 $$\begin{array}{c}\begin{array}{c}{x}_{si}\sim {N}\left({\mu }_{x},{\sigma }_{x}^{2}\right),\\ {e}_{wsi}\sim {N}\left(0, {\sigma }_{ws}^{2}\right),\\ {w}_{si}={a}_{s}+{b}_{s}{x}_{si}+{e}_{wsi}.\end{array}\end{array}$$

This construction ensures that the reference measurement ${x}_{si}$ is independent of the calibration error ${e}_{wsi}$, i.e., $\mathrm{Cov}\left({x}_{si}|{e}_{wsi}\right)=0$, which is consistent with the calibration assumptions in Sect. "Methods". In each study, we randomly remove the reference measurements for a subset of samples, where ${I}_{si}=1$ indicates that the reference measurement for sample $i$ in study $s$ is observed and ${I}_{si}=0$ otherwise. Across all scenarios, we set that ${\mu }_{x}=0$, and study-specific calibration parameters $\left({a}_{1},{a}_{2}, {a}_{3},{a}_{4}\right)=\left(-3, 1,-\mathrm{3,3}\right)$ and $\left({b}_{1},{b}_{2}, {b}_{3},{b}_{4}\right)=\left(0.5, 0.75, 1.25, 1.5\right)$, respectively [5]. For each parameter configuration, we conducted 1000 repeated simulations.

#### Generation of survival outcomes and censoring

For each study, we first generated a linear predictor ${\eta }_{si}$ as defined in Eq. (6). Conditional on ${\eta }_{si}$, event times ${T}_{si}$ were sampled from either an exponential or a Weibull model. In the exponential setting, we specified a constant hazard

41 $$\begin{array}{c}{h}_{si}\left(t|{\eta }_{si}\right)={e}^{{\eta }_{si}},\end{array}$$

such that

42 $$\begin{array}{c}{S}_{si}\left(t|{\eta }_{si}\right)=\mathrm{exp}\left(-{e}^{{\eta }_{si}}t\right).\end{array}$$

In the Weibull setting, we added a shape parameter $\rho>0$ and used a hazard function

43 $$\begin{array}{c}{h}_{si}\left(t|{\eta }_{si}\right)=\rho {t}^{\rho -1}{e}^{{\eta }_{si}},\end{array}$$

which implies the survival function

44 $$\begin{array}{c}{S}_{si}\left(t|{\eta }_{si}\right)=\mathrm{exp}\left(-{e}^{{\eta }_{si}}{t}^{\rho }\right).\end{array}$$

Independent censoring was imposed by sampling censoring times from a uniform distribution

45 $$\begin{array}{c}{C}_{si}\sim Unif\left(0,\uptau \right),\end{array}$$

independent of ${T}_{si}$. The upper bound $\tau$ was chosen to achieve a pre-specified overall censoring rate. For a given $\tau$ and subject-specific hazard rate ${h}_{si}\left(t|{\eta }_{si}\right)$, the theoretical probability of censoring in the exponential model is

46 $$\begin{array}{c}Pr\left({T}_{si}\ge {C}_{si}|{e}^{{\eta }_{si}}\right)=\frac{1-\mathrm{exp}\left(-{e}^{{\eta }_{si}}\uptau \right)}{\uptau {e}^{{\eta }_{si}}},\end{array}$$

and we determined $\tau$ numerically by matching the average censoring probability with the pre-specified overall censoring rate. In the Weibull model, the theoretical censoring probability is

47 $$\begin{array}{c}Pr\left({T}_{si}\ge {C}_{si}|{e}^{{\eta }_{si}}\right)=\frac{1}{\tau \rho \mathrm{exp}\left(\frac{{\eta }_{si}}{\rho }\right)}\gamma \left(\frac{1}{\rho },{e}^{{\eta }_{si}}{\uptau }^{\rho }\right),\end{array}$$

where $\gamma \left(\frac{1}{\rho },{e}^{{\eta }_{si}}{\uptau }^{\rho }\right)$ denotes the lower incomplete gamma function, and $\tau$ was selected analogously to achieve the target censoring rate. The observed follow-up time and event indicator were then defined as

48 $$\begin{array}{c}{t}_{si}=\mathrm{min}\left({T}_{si},{C}_{si}\right),\\{\delta }_{si}=I\left({T}_{i}\le {C}_{i}\right).\end{array}$$

#### Simulation scenarios

In this study, we considered 11 simulation scenarios, as detailed in Table 1. Scenarios 1–5 assumed exponential survival, and Scenarios 6–11 assumed Weibull survival, with four pooled studies in each scenario ($S=4$). For Scenarios 1–11, we varied the true effect size with hazard ratio (HR) values ranging from 1.5 to 2.5, with a step size of 0.25. For exponential scenarios (Scenarios 1–5), we considered per-study sample sizes of 50, 100, and 200 (${n}_{s}=\mathrm{50,100,200}$). For Weibull scenarios (Scenarios 6–11), we fixed ${n}_{s}=100$. We set the Weibull shape parameter $\rho$ = 0.5, 1.0, and 1.5 in Scenario 6–10 to represent decreasing, constant, and increasing hazards, respectively, and fixed $\rho$ =1.0 in Scenario 11.

**Table 1:** Simulation settings of different scenarios and corresponding results for survival outcomes

Scenario Number	Distribution	Rho ($\rho$)	Sample size for each study (${n}_{s}$)	Proportion of samples with reference measurements	Studies without reference measurements $K$/Pooled studies $S$	Censoring rate	Variance (${\sigma }_{x}^{2} \& {\sigma }_{e}^{2}$)	Results in paper
1 (baseline scenario)	Exponential	-	50, 100, 200	0.2	0/4	0.1, 0.3	1, 1	Figure 1, Figure S1-S2, Table S1
2 (high censoring)	Exponential	-	50, 100, 200	0.2	0/4	0.5, 0.7	1, 1	Figure 3, Figure S4-S5, Table S3
3 (sparser calibration subset)	Exponential	-	50, 100, 200	0.1	0/4	0.1, 0.3	1, 1	Figure 5, Figure S7-S8, Table S5
4 (high scale)	Exponential	-	50, 100, 200	0.2	0/4	0.1, 0.3	5, 5	Figure S10-S12, Table S7
5 (high noise)	Exponential	-	50, 100, 200	0.2	0/4	0.1, 0.3	3, 5	Figure S13-S15, Table S8
6 (baseline scenario)	Weibull	0.5, 1, 1.5	100	0.2	0/4	0.1, 0.3	1, 1	Figure 2, Figure S3, Table S2
7 (high censoring)	Weibull	0.5, 1, 1.5	100	0.2	0/4	0.5, 0.7	1, 1	Figure 4, Figure S6, Table S4
8 (sparser calibration subset)	Weibull	0.5, 1, 1.5	100	0.1	0/4	0.1, 0.3	1, 1	Figure 6, Figure S9, Table S6
9 (high scale)	Weibull	0.5, 1, 1.5	100	0.2	0/4	0.1, 0.3	5, 5	Figure S16-S17, Table S9
10 (high noise)	Weibull	0.5, 1, 1.5	100	0.2	0/4	0.1, 0.3	3, 5	Figure S18-S19, Table S10
11 (incomplete calibration structure)	Weibull	1	100	0.2 in studies with reference; 0 otherwise	1/4, 2/4, 3/4	0.1,0.3	1, 1	Table 2, Table S11

Baseline settings were defined as Scenario 1 and Scenario 6, with a reference measurement proportion of 0.2 and censoring rates of 0.1 and 0.3. Scenarios 2–3 and 7–8 examined greater information loss by increasing censoring (0.5 and 0.7) or reducing the reference proportion to 0.1 (sparser calibration). Scenarios 4–5 and 9–10 varied biomarker variance components to assess robustness to increased biological variability and measurement error. Scenarios 4 and 9 increased both the biological variance of ${x}_{si}$ and the measurement error variance between ${x}_{si}$ and ${w}_{si}$, while Scenarios 5 and 10 further considered an extreme case where measurement error variance exceeded biological variance.

Scenario 11 assessed structural missingness of reference measurements, where some contributing studies had no reference measurements. We fixed $\rho =1.0$, considered censoring rates of 0.1 and 0.3. Among the four pooled studies, we set $K$ = 1, 2, or 3 studies to have no reference measurements. Specifically, the no-reference studies were fixed as Study 2 ($K$ = 1), Studies 2–3 ($K$ = 2), and Studies 2–4 ($K$ = 3) across replicates. For studies with reference measurements, the reference sampling proportion was 0.2 as in the baseline setting, whereas it was set to 0 for studies without reference measurements. This scenario mimics common MRCT bridging settings in which reference assays are unavailable in some centers due to logistical or cost constraints.

Across simulation scenarios, different sets of methods were evaluated according to the assumed survival distribution. In Scenarios 1–5, we compared the naive, x-only, two-stage, and VIBPe estimators. In Scenarios 6–11, we additionally evaluated the Weibull-based VIBP estimators VIBPwg and VIBPwl, which differ in their prior specification for $\rho$ (gamma or lognormal). A detailed description of these methods is provided in Sect. "Methods". All simulation scenarios were fitted using the same default algorithmic settings (see Supplementary Section S.C for details).

### Performance measure

We evaluate the methods using four performance measures: bias, standard error (SE), mean squared error (MSE), and coverage rate [28]. Throughout the simulation evaluation, the model is fitted on the log-hazard scale, with effect size denoted by $\beta$ ($HR=\mathrm{exp}(\beta )$). Although simulation scenarios are indexed by true effect values on the HR scale for interpretability, all performance measures below (Bias, SE, MSE, and coverage rate) are computed for $\beta$ on the log-HR scale. Let $\beta$ denote the true effect size, and let ${\widehat{\beta }}_{j}$ denote the estimate from simulation replicate $j,j=1,\dots ,1000$.

#### Bias

For each method and each simulation replicate, we define estimation error (bias for replicate $j$) as

49 $$\begin{array}{c}{\mathrm{Bias}}_{j}={\widehat{\beta }}_{j}-\beta. \end{array}$$

The mean bias was then defined as the average of ${\mathrm{Bias}}_{j}$ across all replicates,

50 $$\begin{array}{c}\mathrm{Mean} \mathrm{bias}=\frac{1}{1000} \sum\limits_{j=1}^{1000}{\mathrm{Bias}}_{j}.\end{array}$$

#### Standard error

The SE is the standard deviation of the estimates across simulation replicates. It is calculated as

51 $$\begin{array}{c}\mathrm{SE}=\sqrt{\frac1{1000-1}\sum\limits_{j=1}^{1000}\left({\widehat\beta}_j-\overline\beta\right)^2},\end{array}$$

where

52 $$\begin{array}{c}\overline{\beta }=\frac{1}{1000} \sum\limits_{j=1}^{1000}{\widehat{\beta }}_{j}\end{array}$$

is the mean estimate.

#### Mean squared error

The MSE is a useful measure of the overall accuracy of a method as it includes both measures of bias and the variability of estimates. The MSE is calculated as

53 $$\begin{array}{c}\mathrm{MSE}=\frac{1}{1000} \sum\limits_{j=1}^{1000}{\left({\widehat{\beta }}_{j}-\beta \right)}^{2}.\end{array}$$

#### Coverage rate

For each method, a 95% confidence interval (CI) for $\beta$ is constructed in every simulation replicate. The coverage probability is defined as the proportion of simulation replicates whose 95% CI covered the true value. A method with good performance should have coverage close to 95%, indicating that around 95% of the confidence intervals include the true value.

### Simulation results

In interpreting the simulation results below, it is helpful to note that increases in MSE may reflect either bias, when estimates systematically shift away from the true effect, or increased dispersion under information loss. Likewise, departures from nominal coverage may arise from bias-induced center shifts or confidence intervals that are too narrow relative to the sampling variability.

#### Results for baseline scenarios

**Fig. 1:**
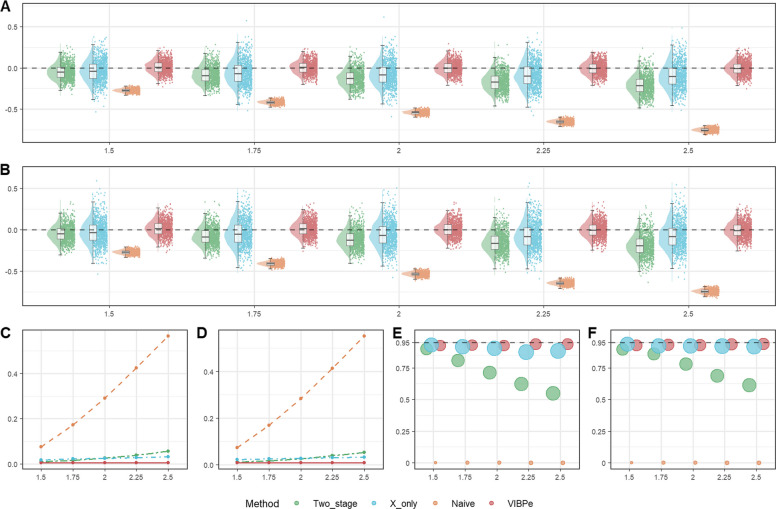
Comparison of operating characteristics under Scenario 1 (exponential baseline scenario) with per-study sample size ${n}_{s}=100$ for the proposed VIBPe, naive, x-only, and two-stage methods. **A** Bias under censoring rate 0.1; **B** Bias under censoring rate 0.3; **C** MSE under censoring rate 0.1; **D** MSE under censoring rate 0.3; **E** Coverage rate under censoring rate 0.1; **F** Coverage rate under censoring rate 0.3. The true effect is indexed on the HR scale ($HR=\mathrm{exp}(\beta )$) for presentation, whereas Bias, MSE, and coverage are computed for $\beta$ on the log-HR scale

**Fig. 2:**
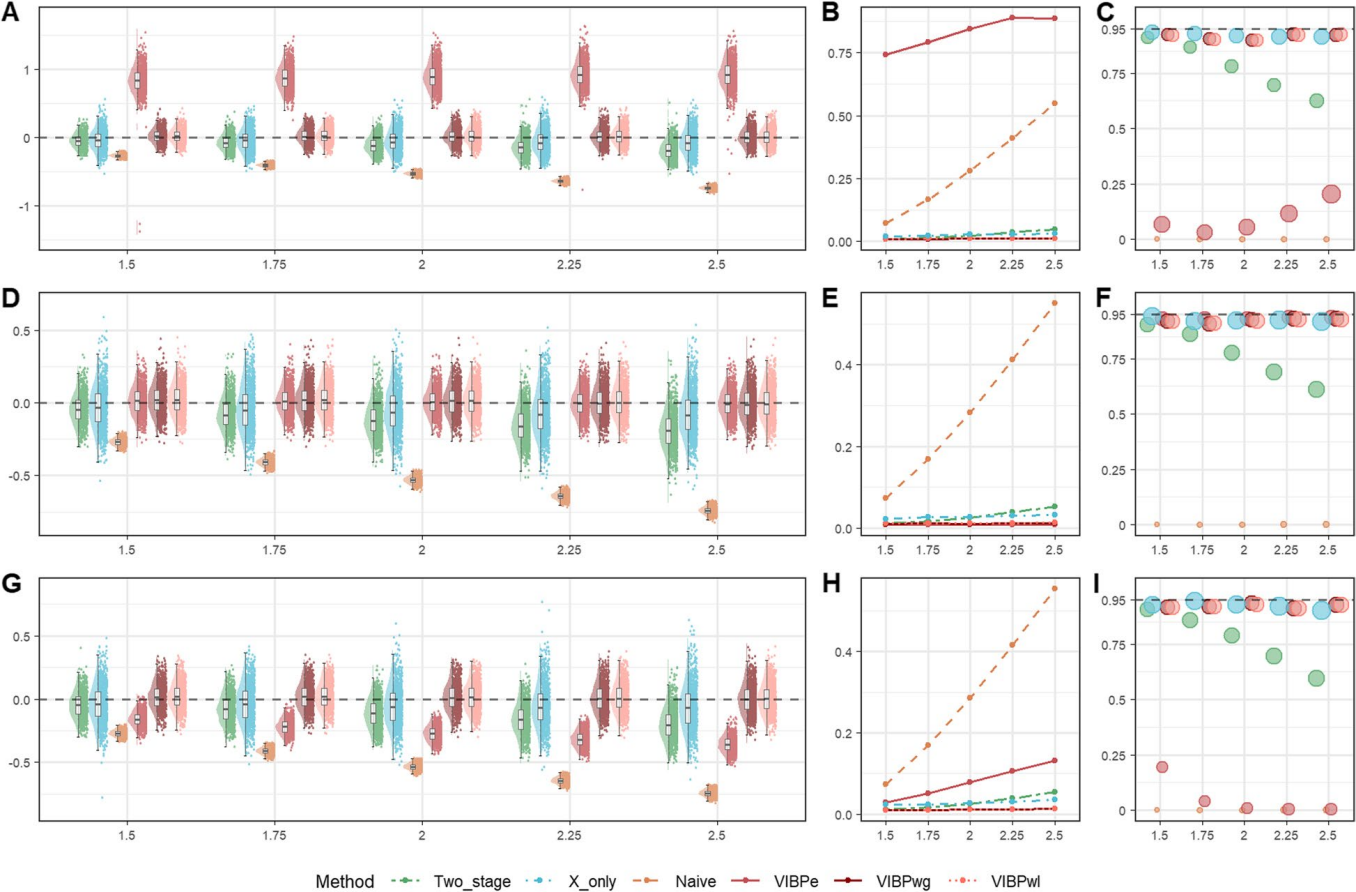
Comparison of operating characteristics under scenario 6 (Weibull baseline scenario) with censoring rate 0.3 for the VIBPe, VIBPwg, VIBPwl, naive, x-only, and two-stage methods. **A** Bias under $\rho$=0.5; **B** MSE under $\rho$=0.5; **C** Coverage rate under $\rho$=0.5; **D** Bias under $\rho$=1.0; **E** MSE under $\rho$=1.0; **F** Coverage rate under $\rho$=1.0; **G** Bias under $\rho$=1.5; **H** MSE under $\rho$=1.5; **I** Coverage rate under $\rho$=1.5. The true effect is indexed on the HR scale ($HR=\mathrm{exp}(\beta )$) for presentation, whereas Bias, MSE, and coverage are computed for $\beta$ on the log-HR scale

We first present the results from the baseline simulation scenarios, namely, Scenario 1 for exponential survival and Scenario 6 for Weibull survival. In Scenario 1, Fig. 1 shows the results for${n}_{s}=100$, and Figures S1-S2 present the corresponding results for ${n}_{s}=50$ and${n}_{s}=200$. Detailed numerical summaries are provided in Table S1. In Scenario 6, Fig. 2 shows the results for a censoring rate of 0.3, and Figure S3 presents the corresponding results for a censoring rate of 0.1. The complete numerical results are presented in Table S2. In these figures, bias values (${\mathrm{bias}}_{j}$) are computed from 1,000 simulation replicates on the log-HR scale for $\beta$, with true effects indexed on the HR scale (i.e.,$HR=\mathrm{exp}(\beta )$), and in the coverage panels the circle sizes are proportional to the average width of the 95% confidence intervals for each method and effect size.

##### Exponential distribution for survival outcome

In Fig. 1, across all effect sizes considered (true HR of 1.5-2.5) and both censoring levels (0.1 and 0.3), VIBPe produces estimates that are tightly centered around the true value (Fig. 1A and B). Its MSE remains small and increases only slightly as the true effect size grows (Fig. 1C and D). The coverage rate is consistently close to the nominal 95%, and the circle sizes in Fig. 1E and F indicate confidence intervals of moderate width. The x-only and two-stage methods exhibit a small negative bias, which becomes more pronounced as the true effect increases. Their estimates are more variable than those from VIBPe, with x-only showing the greatest dispersion. Their MSE values are correspondingly higher and increase gradually with the true effect (Fig.1C and D). The circles for the x-only method are noticeably larger, reflecting wider confidence intervals. For the two-stage method, coverage deteriorates markedly as the true effect increases (from 90.1% to 54.7% as the effect size increases when censoring rate equals 0.1). In contrast, the naive method, which fits a Cox model using only the error-prone local measurements ${w}_{si}$, exhibits strong attenuation toward zero. The bias becomes more severe as the true effect increases, leading to the largest MSE and coverage close to zero.

Across per-study sample sizes (${n}_{s}$= 50, 100, 200), the relative ranking of methods is unchanged. As expected, larger ${n}_{s}$ yields narrower bias distributions and smaller MSE for all methods, with the most pronounced improvements for x-only and two-stage. In contrast, VIBPe shows stable bias and near-nominal coverage across ${n}_{s}$, indicating good finite-sample robustness. 

##### Weibull distribution for survival outcome

In Fig. 2, across all three shape settings, the two Weibull-based VIBP estimators (VIBPwg and VIBPwl) perform consistently well. Their estimates are tightly centered around the true value, with small MSE that increases only mildly as the true effect grows, and coverage rates close to the nominal 95% level with confidence intervals of moderate width. The results for VIBPwg and VIBPwl are very similar, indicating that the choice between a gamma or lognormal prior for $\rho$ has little impact in this baseline setting. This finding suggests that the Weibull-based VIBP approaches are robust to the prior specification for $\rho$. These patterns are consistent across both censoring rates (0.3 in Fig. 2 and 0.1 in Figure S3).

By contrast, the performance of VIBPe depends strongly on the value of $\rho$. When $\rho =1.0$, VIBPe behaves as expected because the Weibull model reduces to the exponential case. That is, the estimates are nearly unbiased, the MSE is small, and coverage is close to 95%. Its performance is slightly better than that of the VIBPwg and VIBPwl estimators. This is reasonable, as VIBPe does not need to estimate the additional shape parameter. However, when $\rho =0.5$, VIBPe shows a clear positive bias and noticeably greater dispersion, leading to inflated MSE. When $\rho =1.5$, the bias becomes negative and again non-negligible, with a corresponding increase in MSE. Thus, VIBPe performs best when the exponential assumption holds ($\rho =1$), but its accuracy deteriorates when the hazard decreases or increases over time, whereas VIBPwg and VIBPwl remain stable across all three shape parameters.

The x-only and two-stage estimators exhibit a small negative bias under all three values of $\rho$, which becomes more pronounced as the true effect increases. Their estimates are more dispersed and their MSE is correspondingly higher. The x-only estimator tends to produce wider confidence intervals, while the two-stage estimator shows lower coverage rates for larger effect sizes (for example, 59.5% when HR=2.5 and $\rho$=1.5), reflecting an underestimation of uncertainty. The naive method exhibits strong attenuation toward zero for all combinations of $\rho$ and true effect, yielding the largest MSE and coverage close to zero.

#### Results under increased information loss

**Fig. 3:**
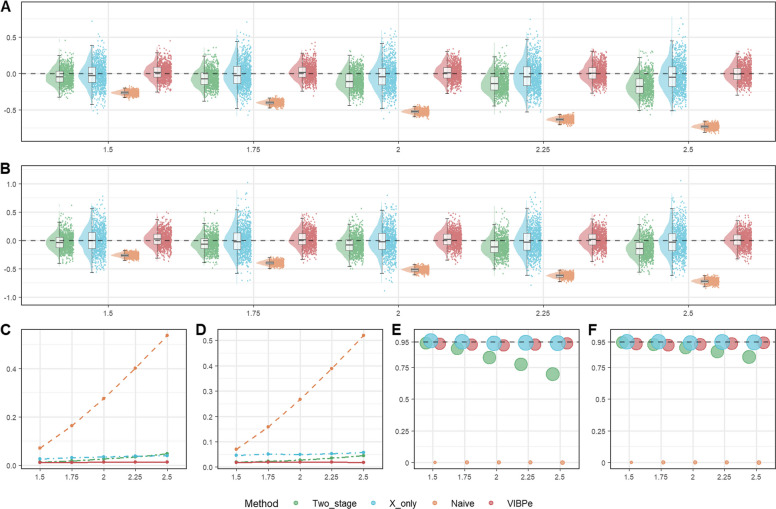
Comparison of operating characteristics under Scenario 2 (exponential high censoring scenario) with per-study sample size ${n}_{s}=100$ for the proposed VIBPe, naive, x-only, and two-stage methods. **A** Bias under censoring rate 0.5; **B** Bias under censoring rate 0.7; **C** MSE under censoring rate 0.5; **D** MSE under censoring rate 0.7; **E** Coverage rate under censoring rate 0.5; **F** Coverage rate under censoring rate 0.7. The true effect is indexed on the HR scale ($HR=\mathrm{exp}(\beta )$) for presentation, whereas Bias, MSE, and coverage are computed for $\beta$ on the log-HR scale

**Fig. 4:**
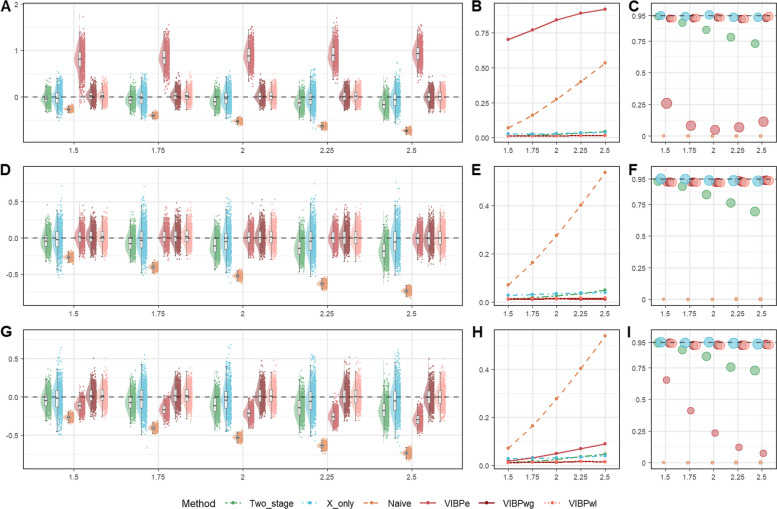
Comparison of operating characteristics under scenario 7 (Weibull high censoring scenario) with censoring rate 0.5 for the VIBPe, VIBPwg, VIBPwl, naive, x-only, and two-stage methods. **A** Bias under $\rho$=0.5; **B** MSE under $\rho$=0.5; **C** Coverage rate under $\rho$=0.5; **D** Bias under $\rho$=1.0; **E** MSE under $\rho$=1.0; **F** Coverage rate under $\rho$=1.0; **G** Bias under $\rho$=1.5; **H** MSE under $\rho$=1.5; **I** Coverage rate under $\rho$=1.5. The true effect is indexed on the HR scale ($HR=\mathrm{exp}(\beta )$) for presentation, whereas Bias, MSE, and coverage are computed for $\beta$ on the log-HR scale

In addition to the baseline scenarios, we considered settings with greater information loss due to higher censoring and sparser calibration data. High censoring levels (0.5 and 0.7) are common in time-to-event trials with long-term endpoints or low event rates, such as adjuvant oncology studies in which many patients remain event-free at the end of follow-up. For exponential survival (Scenario 2), the four methods were evaluated at ${n}_{s}$ = 50, 100, and 200. Figure 3 shows the results for ${n}_{s}=100$, and Figures S4–S5 present the corresponding results for ${n}_{s}=50$ and ${n}_{s}=200$. For Weibull survival with high censoring (Scenario 7), we examined the six methods across $\rho =$ 0.5, 1.0, and 1.5. Figure 4 shows the results for a censoring rate of 0.5, and Figure S6 shows the results for a censoring rate of 0.7. Numerical summaries for these scenarios are provided in Tables S3–S4.

**Fig. 5:**
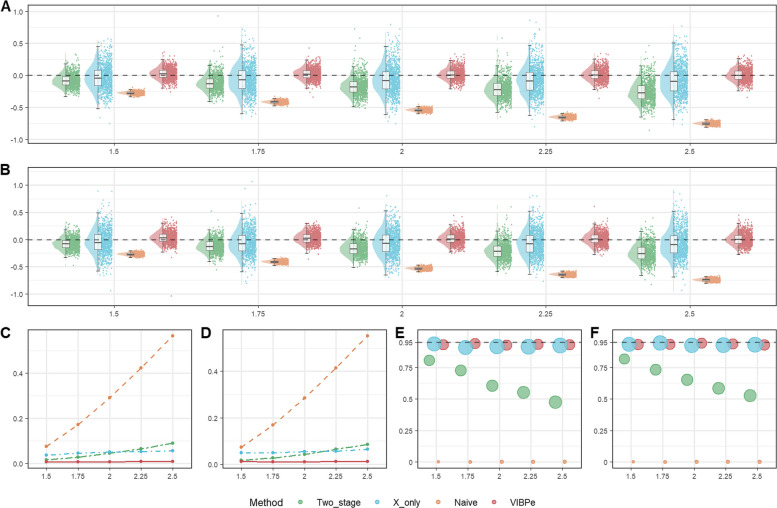
Comparison of operating characteristics under Scenario 3 (exponential sparser calibration subset scenario) with per-study sample size ${n}_{s}=100$ for the proposed VIBPe, naive, x-only, and two-stage methods. **A** Bias under censoring rate 0.1; **B** Bias under censoring rate 0.3; **C** MSE under censoring rate 0.1; **D** MSE under censoring rate 0.3; **E** Coverage rate under censoring rate 0.1; **F** Coverage rate under censoring rate 0.3. The true effect is indexed on the HR scale ($HR=\mathrm{exp}(\beta )$) for presentation, whereas Bias, MSE, and coverage are computed for $\beta$ on the log-HR scale

**Fig. 6:**
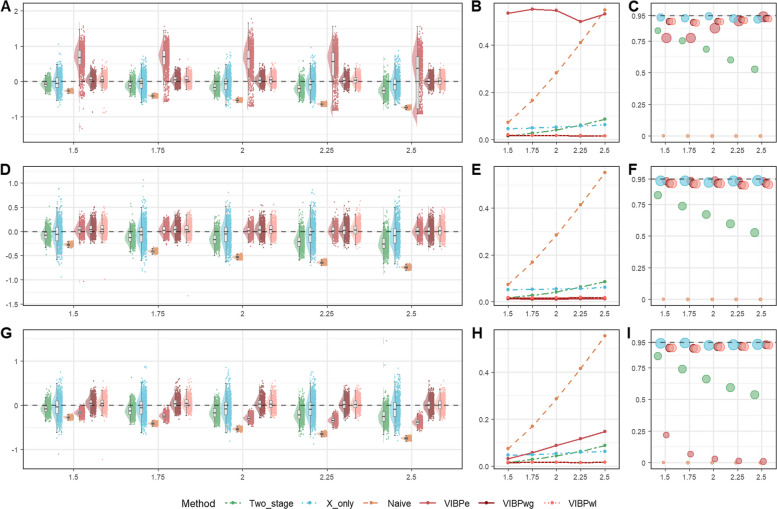
Comparison of operating characteristics under scenario 8 (Weibull sparser calibration subset scenario) with censoring rate 0.3 for the VIBPe, VIBPwg, VIBPwl, naive, x-only, and two-stage methods. **A** Bias under $\rho$=0.5; **B** MSE under $\rho$=0.5; **C** Coverage rate under $\rho$=0.5; **D** Bias under $\rho$=1.0; **E**. MSE under $\rho$=1.0; **F** Coverage rate under $\rho$=1.0; **G** Bias under $\rho$=1.5; **H** MSE under $\rho$=1.5; I. Coverage rate under $\rho$=1.5. The true effect is indexed on the HR scale ($HR=\mathrm{exp}(\beta )$) for presentation, whereas Bias, MSE, and coverage are computed for $\beta$ on the log-HR scale

A reduced proportion of subjects with reference measurements from 0.2 to 0.1 reflects practical funding constraints in clinical research, where the number of samples re-assayed is often determined by available resources. For exponential survival with sparse calibration (Scenario 3), the four methods were again evaluated at ${n}_{s}$ = 50, 100, and 200. Figure 5 shows the results for ${n}_{s}=100$, and Figures S7–S8 show the results for ${n}_{s}=50$ and ${n}_{s}=200$. For the corresponding Weibull setting (Scenario 8), the six methods were compared across $\rho =$ 0.5, 1.0, and 1.5. Figure 6 shows the results for a censoring rate of 0.3, and Figure S9 shows the results for a censoring rate of 0.1. Numerical results for these scenarios are summarized in Tables S5–S6.

##### Exponential survival under higher censoring

Under the higher censoring levels (Fig. 3), the relative ranking of the four methods is similar to that in the baseline scenario. However, the bias distributions become more dispersed and the MSE values generally increase. This dispersion is most evident at smaller ${n}_{s}$, consistent with compounded information loss from censoring and limited sample size. VIBPe still has the smallest bias and MSE and maintains coverage close to the nominal 95% level. These findings suggest that VIBPe remains robust under high censoring for exponential survival outcomes.

##### Weibull survival under higher censoring

Figure4 shows that the overall ordering of methods is similar to the baseline Weibull scenario. Across the three shape parameters $\rho$ = 0.5, 1.0, and 1.5, the three VIBP estimators show more nuanced patterns. When $\rho =1.0$, all three VIBP methods (VIBPe, VIBPwg and VIBPwl) perform well. Their estimates are tightly centered around the true value, MSE is small and coverage is close to the nominal 95% level. When $\rho =0.5$ and 1.5, VIBPwg and VIBPwl still maintain small bias and acceptable coverage, whereas VIBPe shows clear bias, inflated MSE and low coverage rates.

The x-only and two-stage estimates are more dispersed and their MSE is higher than for the better performing VIBP estimators. Coverage rate for the x-only method stays close to 95%, largely due to wider confidence intervals. The two-stage method drops at larger effect sizes. Meanwhile, the naive estimator remains clearly biased toward zero, with large MSE and coverage close to zero. Overall, under high censoring, VIBPwg and VIBPwl estimators continue to provide low bias, low MSE, and near-nominal coverage across the different shape parameters, while VIBPe is less compatible with the data when $\rho =0.5$ or $\rho =1.5$.

##### Exponential survival under reduced proportion of reference measurements

In Fig. 5, the bias distributions for the two-stage, x-only and VIBPe estimators become wider, and their MSE values increase. The mean bias shifts more for the two-stage and for VIBPe. This pattern is consistent with increased reliance on error-prone local measurements ${w}_{si}$ when the calibration subset becomes smaller. With fewer reference observations, the calibration step becomes less precise and the survival model relies more heavily on ${w}_{si}$. In contrast, there is essentially no impact on the naive estimator as expected, because it relies only on the local measurements ${w}_{si}$ without using the reference data. Even in this setting, VIBPe continues to show small bias, low MSE and coverage close to the nominal level, which indicates good robustness when the proportion of reference measurements is low.

##### Weibull survival under reduced proportion of reference measurements

In Fig. 6, the VIBP estimators are also affected, but to a lesser extent. VIBPwg and VIBPwl keep small bias, low MSE and coverage near the nominal level for all three values of $\rho$. VIBPe shows some loss of performance when $\rho =0.5$, with the largest MSE and lower coverage, and a bimodal sampling distribution of the estimates. When$\rho =1.5$, VIBPe again has larger MSE and lower coverage rate, improving only over the naive estimator. The x-only and two-stage estimators become more variable than in the baseline Weibull scenario, and the MSE increases accordingly. Coverage rate for the x-only method remains close to 95%, while the two-stage estimator declines at larger effects. The naive estimator again shows poor performance across all values of $\rho$ and all effect sizes.

#### Results under different variability

Scenarios 4–5 and 9–10 considered settings with larger biomarker variability and higher measurement error. For biomarker data, variability can be substantial. Both the biological variance of ${x}_{si}$ and the measurement error variance between ${x}_{si}$ and ${w}_{si}$ can be large, as is commonly seen for small-molecule metabolites measured by chromatography-mass spectrometry, where signal complexity and batch effects introduce considerable noise despite high instrumental precision.

##### Exponential distribution for survival outcome

For exponential survival (Scenarios 4 and 5), the four methods were evaluated at ${n}_{s}$ = 50, 100, and 200. The corresponding results are shown in Figures S10-S15, with numerical summaries in Tables S7-S8. Overall, increasing the overall scale of ${x}_{si}$ and the measurement error variance leads to slightly wider bias distributions for all methods and modestly larger MSE for two-stage method, but does not change the relative ranking. VIBPe continues to have the smallest MSE and coverage close to the nominal 95% level. The x-only and two-stage estimators are more variable. The naive estimator remains strongly attenuated toward zero with large MSE and almost no coverage.

##### Weibull distribution for survival outcome

For Weibull survival (Scenarios 9 and 10), all six methods were compared across shape parameters $\rho =$ 0.5, 1.0, and 1.5. Results for censoring rates of 0.1 and 0.3 are presented in Figures S16-S19, with numerical summaries in Tables S9-S10. VIBPwg and VIBPwl show small bias, low MSE and coverage close to 95% across the different values of $\rho$, while VIBPe performs very well when $\rho =1.0$ and shows some loss of accuracy when the hazard decreases or increases over time ($\rho =0.5$ or $\rho =1.5$), consistent with the findings from the baseline Weibull scenarios. The x-only and two-stage estimators again exhibit larger variability, whereas the naive estimator continues to perform poorly.

#### Results for structural missingness of reference measurements

Finally, we evaluated structural missingness of reference measurements, where an increasing number of contributing studies had no reference biomarker data (Scenario 11). Table 2 presents results under a censoring rate of 0.3 for HR = 1.5, 2.0, and 2.5, while the full results across HR values (1.5–2.5 in increments of 0.25) and censoring rates of 0.1 and 0.3 are provided in Supplementary Table S11. The naive method exhibits strong attenuation toward the null across all HRs and all values of $K$, where $K$ denotes the number of studies without any reference measurements. In contrast, the VIBP estimators remain close to the truth and provide reliable inference when $K$=1. As $K$ increases to 2 and 3, their performance deteriorates, which is consistent with diminishing calibration information in the pooled data. Among the VIBP estimators, VIBPe is generally the most stable as structural missingness becomes more severe. Overall, VIBP can accommodate study-level structural missingness of reference measurements and still recover interpretable biomarker-survival associations under sparse and heterogeneous calibration information.

**Table 2:** Results under a censoring rate of 0.3 for HR of 1.5, 2.0, and 2.5

HR	Method	$K$=1			$K$=2			$K$=3		
		Bias*100(Se*100)	MSE*100	CR (%)	Bias*100(Se*100)	MSE*100	CR (%)	Bias*100(Se*100)	MSE*100	CR (%)
1.5	VIBPe	2.37(0.30)	0.94	92.1	5.41(0.33)	1.36	87.9	5.48(0.86)	7.62	88.3
	VIBPwg	3.14(0.31)	1.09	91.9	7.09(0.36)	1.80	84.7	10.64(0.91)	9.48	80.7
	VIBPwl	3.39(0.32)	1.12	91.7	7.28(0.38)	1.97	84.3	9.08(1.03)	11.49	80.6
	Naive	−27.05(0.07)	7.37	0	−27.03(0.07)	7.36	0	−27.03(0.07)	7.36	0
2	VIBPe	1.55(0.30)	0.93	92.9	3.30(0.32)	1.16	91.2	5.50(0.61)	3.99	89.8
	VIBPwg	2.46(0.34)	1.22	92	5.10(0.37)	1.61	87.4	9.78(0.67)	5.44	79.7
	VIBPwl	2.89(0.34)	1.27	91.8	5.61(0.37)	1.69	86.9	9.33(0.80)	7.26	80.1
	Naive	−53.29(0.07)	28.46	0	−53.28(0.07)	28.44	0	−53.28(0.07)	28.44	0
2.5	VIBPe	−0.27(0.31)	0.94	93.9	1.02(0.34)	1.18	93	2.26(0.55)	3.11	91.8
	VIBPwg	−0.30(0.36)	1.28	92.7	1.55(0.40)	1.61	92	4.35(0.60)	3.80	88.5
	VIBPwl	0.28(0.36)	1.31	92.4	2.23(0.40)	1.68	91.3	5.52(0.55)	3.37	87.6
	Naive	−74.25(0.08)	55.19	0	−74.23(0.07)	55.15	0	−74.23(0.07)	55.15	0

*CR* Coverage rate. $K$ denotes the number of studies without any reference measurements

## Application and case study

To illustrate the proposed methodology, we analyzed data from The Cancer Genome Atlas (TCGA) lung squamous cell carcinoma (LUSC) cohort to evaluate the association between DJ-1 protein (encoded by PARK7) and overall survival. DJ-1 has been reported to be a clinically relevant cancer-related protein, and circulating or secreted DJ-1 has been linked to tumor progression and patient outcomes in lung cancer and other malignancies [29, 30]. DJ-1 protein expression measured by reverse-phase protein array was treated as the reference biomarker, and PARK7 gene expression derived from RNA sequencing was treated as an upstream proxy with measurement noise. No additional transformation or standardization was applied to the biomarker variables beyond the values provided by the UCSC Xena TCGA resource. Due to cost and platform constraints, protein measurements were available only for a subset of patients, whereas gene expression data were broadly observed. This resulted in a calibration-based survival analysis setting with systematically missing reference biomarkers (Sect. "Methods"), which were handled through the model’s latent-reference structure rather than imputed.

To evaluate robustness under multi-study integration, we combined data from five sampling centers. The total sample sizes per center were 50, 44, 40, 39, and 37, respectively, whereas only 12, 12, 6, 21, and 22 patients per center had observed protein data. This imbalance led to sparse and heterogeneous calibration data at the center level, posing challenges for conventional regression calibration approaches and motivating the proposed joint hierarchical modeling strategy (VIBP).

Across center combinations, both Weibull-based VIBP variants yielded consistent positive log-hazard ratio estimates for DJ-1, with point estimates around 0.60 and confidence intervals excluding 0 (Table 3). The exponential VIBP estimator provided a similar result. In contrast, the x-only and two-stage approaches produced comparable point estimates but wider intervals that crossed 0, which was consistent with limited effective sample size and unstable center-specific calibration. The naive approach gave a markedly smaller estimate, which was consistent with attenuation when an upstream proxy was used without explicit measurement-error correction. These findings demonstrate that VIBP can provide stable survival effect estimation when the reference biomarker is partially observed and calibration information is sparse, while maintaining internal consistency across model specifications.

**Table 3:** Point and 95% confidence interval (CI) estimates for the association of DJ1 and LUSC using TCGA data

Method	${\widehat{{\boldsymbol{\beta}}}}_{{\boldsymbol{x}}}$	HR	HR 95%CI
Two-stage	0.7057	2.0254	(0.7316,5.6068)
X-only	0.7092	2.0324	(0.9770,4.2278)
Naive	0.2519	1.2865	(0.8935,1.8525)
VIBPe	0.5711	1.7701	(1.0416,3.2121)
VIBPwg	0.6012	1.8243	(1.0176,3.9579)
VIBPwl	0.6042	1.8299	(1.0167,4.0008)

## Discussion

Across a wide range of simulation scenarios, the proposed VIBP methods provided accurate and robust estimation in the presence of inter-study variability and partial observation of reference measurements. Importantly, VIBP remains applicable even under study-level structural missingness of reference measurements, which is a common feature of MRCT bridging and multi-study integration settings. For exponential survival data, VIBPe yielded estimates with small bias, the lowest MSE, and coverage close to the nominal 95% level across different effect sizes, censoring rates, sample sizes, and variance configurations. For Weibull survival data, the two Weibull-based estimators (VIBPwg and VIBPwl) showed consistently good performance over all shape parameters considered. In contrast, VIBPe performed well only when the hazard was approximately constant ($\rho$=1.0) and deteriorated when the hazard was non-constant ($\rho$=0.5 or 1.5). The naive estimator was severely attenuated toward zero with large MSE and poor coverage rate. The x-only and two-stage estimators reduced bias but remained more variable and tended to under-cover at larger effect sizes. The real data application based on TCGA LUSC further illustrated these patterns in a realistic setting with sparse calibration data and substantial measurement error. For the DJ-1 protein encoded by PARK7, VIBP identified a statistically significant association with overall survival and produced stable effect estimates. However, the x-only and two-stage methods exhibited wide confidence intervals, and the naive estimator showed clear attenuation. The consistency of VIBP estimates supports its ability to recover meaningful survival associations.

These results can be explained by how each method uses the available information. The naive method fits a survival model directly to the error-prone local measurements, which induces systematic attenuation bias, markedly larger MSE, and coverage rates close to zero. The x-only method avoids this bias by restricting analysis to subjects with reference measurements but substantially reduces the effective sample size, leading to dispersed estimates across simulation replicates and wider 95% CIs. The two-stage method incorporates both reference and local measurements but relies on regression calibration through a ratio estimator. This can be unstable under small sample sizes and substantial measurement error, introducing additional variability and nonlinear bias. The VIBP treats the unobserved true biomarker values as latent variables and models both the calibration relationship and the survival outcome within a single unified Bayesian joint model. The coherent likelihood-based formulation avoids the information loss of the x-only method and the instability of ratio-type corrections in the two-stage approach. The variational inference framework yields regularized estimates that integrate information through the shared latent biomarker, resulting in improved stability.

Several limitations merit consideration. For Weibull models, we set ${{q}}\left({{\rho}}\right)$ to a Dirac delta at the value that maximizes the ELBO with respect to ${{\rho}}$ conditional on the other variational factors. This strategy improves numerical stability, but it also treats ${{\rho}}$ as a point estimate and therefore does not fully capture uncertainty in ${{\rho}}$. Under a lognormal prior, this one-dimensional objective is not globally concave. Although it was typically well-behaved near the optimum, challenges may persist in small or weakly informative samples. We recommend the gamma prior as a default choice due to its more reliable numerical behavior, but further work is needed to develop more flexible and stable approximations for ${{\rho}}$ that better capture uncertainty while maintaining robust convergence, for example by using richer variational families. In a small fraction of simulation replicates, VIBPe produced extreme estimates. These outlying solutions occurred primarily in low-information settings (few events, high censoring, and limited calibration data), where the constant-hazard assumption may impose undue constraints. When the true hazard varies over time, the model may compensate by allocating lack-of-fit to the regression component or latent biomarker layer, leading to boundary solutions or convergence to suboptimal local optima. In contrast, the Weibull-based variants (VIBPwg and VIBPwl) introduce additional flexibility through ${{\rho}}$, which can absorb time-varying risk patterns and reduce compensatory distortion in the regression coefficients. Accordingly, we recommend VIBPwg as the default, and use VIBPe when hazards are approximately constant or as a sensitivity analysis. More generally, extending the framework to accommodate more flexible baseline hazards beyond the Weibull family and developing practical model-checking diagnostics would further strengthen robustness in applications. Finally, our simulations focused on linear calibration models, consistent with the assumed data-generating mechanism. In practice, calibration relationships may be nonlinear, heteroscedastic, or study dependent in more complex ways. Assessing robustness to calibration model misspecification and extending the framework to more flexible calibration structures remain important directions for future research.    

## Conclusions

In this study, we propose a unified Bayesian joint model that provides accurate and robust estimation under inter-study measurement heterogeneity and partially observed reference measurements, and remains applicable when some studies have no reference data.

## Supplementary Information

Supplementary Material 1.

Supplementary Material 2.

## Data Availability

The simulation code and analysis pipeline (including scripts to reproduce the main results) are available online at [https://github.com/luyiyun/VIBPs]. The real-data case study is based on publicly available TCGA Lung Squamous Cell Carcinoma (LUSC) data, which can be downloaded from the UCSC Xena browser at [https://xenabrowser.net/datapages/?cohort=TCGA%20Lung%20Squamous%20Cell%20Carcinoma%20(LUSC)&removeHub=https%3A%2F%2Flocal.xena.ucsc.edu%3A7223]. We did not generate new human-subject data and do not redistribute individual-level data.

## Abbreviations

VIBP: Variational Inference-based Biomarker Pooling
CAVI: Coordinate Ascent Variational Inference
HR: Hazard Ratio
SE: Standard Error
MSE: Mean Squared Error
CR: Coverage Rate
TCGA: The Cancer Genome Atlas
LUSC: Lung Squamous Cell Carcinoma
