# Supplementary material for: Variational biomarker pooling with calibration for time-to-event outcomes across multiple clinical studies
Source: BMC Med Res Methodol. 2026 Mar 23;26:97. doi: 10.1186/s12874-026-02827-y (PMC13130471; doi:10.1186/s12874-026-02827-y)
Supplement: Supplementary file 2 — Supplementary Material 2. [file 12874_2026_2827_MOESM2_ESM.pdf]

Supplementary Files

CONTENT

S.A Derivations of the variational updates **2**

S.A.1 Notation and general CAVI update 2

S.A.2 Variational posterior for the outcome parameters $\theta\beta$ 3

S.A.3 Variational posterior for the study-specific parameters $us, \sigma ws2$ 4

S.A.4 Variational posterior for the latent biomarkers xsi (for $Isi = 0$) 6

S.A.5 Variational posterior for $\mu x, \sigma x2$ 7

S.B Variational Gamma approximation for the Weibull shape parameter **9**

S.C Implementation details **12**

S.D Comparison of operating characteristics under multiple scenarios **13**

S.E Schematic figure **32**

S.A Derivations of the variational updates

This section provides the main derivations of the mean-field variational updates summarized in Equations (17)-(23) of the main text. For clarity, we focus on the exponential survival model (VIBPe); the Weibull-based variants (VIBPwg and VIBPwl) share the same algebraic structure, with the Poisson weights ω_{si} adjusted as described in Section 2.3.

S.A.1 Notation and general CAVI update

Under the mean-field factorization in Equation (14),

$$q(\theta, x) = q(\theta_\beta) \prod_{s=1}^{S} q(u_s) q(\sigma_{ws}^2) \prod_{(s,i):I_{si}=0} q(x_{si})\, q(\mu_x, \sigma_x^2), \qquad (S.A.1)$$

the evidence lower bound (ELBO) is

$$\mathcal{L}(q) = E_q[\log p\,(y, w, x, \theta \mid Z)] - E_q[\log q\,(x, \theta)]. \qquad (S.A.2)$$

For any block θ_j (or latent variable), the CAVI update is

$$\log q^*(\theta_j) = E_{q(\theta_{-j,x})}[\log p(y, w, x, \theta)] + \text{const}, \qquad (S.A.3)$$

where θ_{-j} collects all parameters and latent variables except θ_j, and "const" denotes terms that do not depend on θ_j. We use an IRLS-based quadratic surrogate for the outcome likelihood. For the exponential model (Section 2.2),

$$l_{si}(\eta_{si}) \approx -\frac{1}{2}\omega_{si}(\eta_{si} - z_{si}^*)^2 + \text{const}, \omega_{si} = \bar{\mu}_{si} = t_{si} E(e^{\eta_{si}}), \qquad (S.A.4)$$

and for Weibull model (Section 2.3),

$$l_{si}(\eta_{si}; \rho) \approx -\frac{1}{2}\omega_{si}(\eta_{si} - z_{si}^*)^2 + \text{const}, \omega_{si} = \bar{\mu}_{si} = t_{si}{}^{\rho} E(e^{\eta_{si}}). \qquad (S.A.5)$$

In both cases, $z_{si}^* = m_{\eta,si} + \frac{\delta_{si} - \bar{\mu}_{si}}{\bar{\mu}_{si}}$, and the surrogate is quadratic in the linear predictor η_{si}, which is key to obtaining Gaussian variational factors.

S.A.2 Variational posterior for the outcome parameters $\boldsymbol{\theta_\beta}$

Recall that $\eta_{si} = \psi_{si}^T \theta_\beta$ and $\psi_{si} = (e_s^T, x_{si}, z_{si}^T)^T$, where the e_s is the *S*-dimensional unit vector with a '1' in the position corresponding to study s, and x_{si} is either the observed x_{si} or the current variational mean $E_q[x_{si}]$. Using the Equation (S.A.4), the approximate contribution of the outcome likelihood to the joint log-density is

$$\sum_{s,i} l_{si}(\eta_{si}) \approx -\frac{1}{2}\sum_{s,i} \omega_{si}\left(\psi_{si}^T\theta_\beta - z_{si}^*\right)^2 + \text{const}$$

$$= -\frac{1}{2}\theta_\beta^T\left(\sum_{s,i}\omega_{si}\,\psi_{si}\psi_{si}^T\right)\theta_\beta + \theta_\beta^T\left(\sum_{s,i}\omega_{si}z_{si}^*\,\psi_{si}\right) + \text{const.} \qquad (S.A.6)$$

The prior for $\boldsymbol{\theta_\beta}$ is multivariate normal, so its log-density is

$$\log p(\theta_\beta) = -\frac{1}{2}\theta_\beta^T\left(\Sigma_\beta^0\right)^{-1}\theta_\beta + \theta_\beta^T\left(\Sigma_\beta^0\right)^{-1}m_\beta^0 + \text{const.} \qquad (S.A.7)$$

Substituting Equations (S.A.6)-(S.A.7) into the generic update Equation (S.A.3) with $\theta_j = \theta_\beta$, and taking expectations with respect to all variational factors except $q(\theta_\beta)$, we obtain

$$\log q^*(\theta_\beta) = E_q\left[\sum_{s,i} l_{si}(\eta_{si}) + \log p(\theta_\beta)\right] + \text{const}$$

$$= -\frac{1}{2}\theta_\beta^T\left(\sum_{s,i}\omega_{si}\,E_q[\psi_{si}\psi_{si}^T] + \left(\Sigma_\beta^0\right)^{-1}\right)\theta_\beta + \theta_\beta^T\left(\sum_{s,i}\omega_{si}z_{si}^*E_q[\psi_{si}] + \left(\Sigma_\beta^0\right)^{-1}m_\beta^0\right) + \text{const} \qquad (S.A.8)$$

The right-hand side is the log of a multivariate normal density. Matching it to the canonical quadratic form, we identify

$$q(\theta_\beta) = \mathcal{N}(m_\beta, \Sigma_\beta), \tag{S.A.9}$$

with

$$\Sigma_\beta = \left(\sum_{s,i} \omega_{si}\, E_q[\psi_{si}\psi_{si}^T] + \left(\Sigma_\beta^0\right)^{-1}\right)^{-1},$$

$$m_\beta = \Sigma_\beta \left(\sum_{s,i} \omega_{si} z_{si}^* E_q[\psi_{si}] + \left(\Sigma_\beta^0\right)^{-1} m_\beta^0\right),$$

which corresponds to Equation (17) in the main text.

S.A.3 Variational posterior for the study-specific parameters $(\boldsymbol{u}_s, \boldsymbol{\sigma}_{ws}^2)$

Let $u_s = (a_s, b_s)^T$ and $\Phi_{si} = (1, x_{si})^T$. The calibration model is

$$w_{si} | x_{si}, u_s, \sigma_{ws}^2 = \mathcal{N}(u_s^T \Phi_{si}, \sigma_{ws}^2). \tag{S.A.10}$$

The corresponding log-likelihood contribution for study s is

$$\sum_i \log p(w_{si} | u_s, \sigma_{ws}^2, x_{si}) = -\frac{n_s}{2} \log \sigma_{ws}^2 - \frac{1}{2\sigma_{ws}^2} \sum_i (w_{si} - u_s^T \Phi_{si})^2 + \text{const.} \tag{S.A.11}$$

S.A.3.1 Update for $\boldsymbol{q}(\boldsymbol{u}_s)$

The prior for u_s is multivariate normal, so its log-density is

$$\log p(u_s) = -\frac{1}{2} u_s^T (\Sigma_{ab}^0)^{-1} u_s + u_s^T (\Sigma_{ab}^0)^{-1} \mu_{ab}^0 + \text{const.} \tag{S.A.12}$$

Applying Equation (S.A.3) with $\theta_j = u_s$ and keeping only terms involving u_s,

$$\log q^*(u_s) = E_{q(\sigma_{ws}^2,x)}\left[-\frac{1}{2\sigma_{ws}^2}\sum_i (w_{si} - u_s^T\Phi_{si})^2 + \log p(u_s)\right] + \text{const}$$

$$= -\frac{1}{2}u_s^T\left(E_q[\sigma_{ws}^{-2}]\sum_i E_q[\Phi_{si}\Phi_{si}^T] + (\Sigma_{ab}^0)^{-1}\right)u_s + u_s^T\left(E_q[\sigma_{ws}^{-2}]\sum_i w_{si}E_q[\Phi_{si}] + (\Sigma_{ab}^0)^{-1}\mu_{ab}^0\right) + \text{const.} \qquad (S.A.13)$$

This is again the log of a multivariate normal distribution, so

$$q(u_s) = \mathcal{N}\big(m_{ab,s}, \Sigma_{ab,s}\big), \qquad (S.A.14)$$

with

$$\Sigma_{ab,s} = \left(E_q[\sigma_{ws}^{-2}]\sum_i E_q[\Phi_{si}\Phi_{si}^T] + (\Sigma_{ab}^0)^{-1}\right)^{-1},$$

$$m_{ab,s} = \Sigma_{ab,s}\left(E_q[\sigma_{ws}^{-2}]\sum_i w_{si}E_q[\Phi_{si}] + (\Sigma_{ab}^0)^{-1}m_{ab}^0\right),$$

as stated in Equation (18) of the main text.

S.A.3.2 Update for $q(\sigma_{ws}^2)$

The prior for σ_{ws}^2 is Inverse-Gamma, whose log-density is

$$\log p(\sigma_{ws}^2) = -(\alpha_w^0 + 1)\log \sigma_{ws}^2 - \frac{\gamma_w^0}{\sigma_{ws}^2} + \text{const.} \qquad (S.A.15)$$

Substituting Equations (S.A.11) and (S.A.15) into the generic update Equation (S.A.3) with $\theta_j = \sigma_{ws}^2$, we obtain

$$\log q^*(\sigma_{ws}^2) = E_{q(u_s,x)}\left[-\frac{n_s}{2}\log \sigma_{ws}^2 - \frac{1}{2\sigma_{ws}^2}\sum_i (w_{si} - u_s^T\Phi_{si})^2 + \log p(\sigma_{ws}^2)\right] + \text{const}$$

$$= -\left(\frac{n_s}{2} + \alpha_w^0 + 1\right) \log \sigma_{ws}^2 - \frac{1}{\sigma_{ws}^2}\left(\frac{1}{2}\sum_{i=1}^{n_s} E_q[(w_{si} - u_s^T \Phi_{si})^2] + \gamma_w^0\right) + \text{const.} \quad (S.A.16)$$

Recognizing the kernel of an Inverse-Gamma distribution, we have

$$q(\sigma_{ws}^2) = \text{InvGamma}(\alpha_{w,s}, \gamma_{w,s}), \quad (S.A.17)$$

with

$$\alpha_{w,s} = \frac{n_s}{2} + \alpha_w^0, \gamma_{w,s} = \frac{1}{2}\sum_{i=1}^{n_s} E_q[(w_{si} - u_s^T \Phi_{si})^2] + \gamma_w^0,$$

which matches Equation (19) in the main text.

S.A.4 Variational posterior for the latent biomarkers x_{si} (for $I_{si} = 0$)

For subjects with missing reference measurements ($I_{si} = 0$), the variational factor $q(x_{si})$ is updated using three model components, that is, the outcome model, the calibration model, and the marginal prior for x_{si}. Recall $\eta_{si} = \beta_{0s} + \beta_x x_{si} + \beta_z^\top z_{si}$, the approximate contribution of the outcome likelihood to the joint log-density is

$$\begin{aligned} l_{si}(\eta_{si}) &\approx -\frac{1}{2}\omega_{si}(\beta_x x_{si} + \beta_{0s} + \beta_z^\top z_{si} - z_{si}^*)^2 + \text{const} \\ &= -\frac{1}{2}\omega_{si}[\beta_x^2 x_{si}^2 + 2\beta_x(\beta_{0s} + \beta_z^\top z_{si} - z_{si}^*)x_{si}] + \text{const.} \end{aligned} \quad (S.A.18)$$

The log-likelihood contribution of calibration model for study s is

$$\begin{aligned} \log p(w_{si}|u_s, \sigma_{ws}^2, x_{si}) &= -\frac{1}{2\sigma_{ws}^2}(w_{si} - a_s - b_s x_{si})^2 + \text{const} \\ &= -\frac{1}{2}\sigma_{ws}^2[b_s^2 x_{si}^2 - 2b_s(w_{si} - a_s)x_{si}] + \text{const.} \end{aligned} \quad (S.A.19)$$

For the marginal prior x_{si}

$$\log p(x_{si}|\mu_x,\sigma_x^2) = -\frac{1}{2\sigma_x^2}(x_{si}-\mu_x)^2$$

$$= -\frac{1}{2}\sigma_x^{-2}x_{si}^2 + \sigma_x^{-2}\mu_x x_{si} + \text{const.} \qquad (S.A.20)$$

Substituting Equations (S.A.18)-(S.A.20) and applying Equation (S.A.3) with $\theta_j = x_{si}$, we obtain a quadratic form

$$\log q^*(x_{si}) = E_q[l_{si}(\eta_{si}) + \log p(w_{si}|u_s,\sigma_{ws}^2,x_{si}) + \log p(x_{si}|\mu_x,\sigma_x^2)] + \text{const}$$

$$= -\frac{1}{2}\left(\omega_{si}E_q[\beta_x^2] + E_q[\sigma_{ws}^{-2}]E_q[b_s^2] + E_q[\sigma_x^{-2}]\right)x_{si}^2 + D_{si}x_{si} + \text{const}, \qquad (S.A.21)$$

where

$$D_{si} = \omega_{si}E_q[\beta_x]\left(z_{si}^* - E_q[\beta_{0s}] - E_q[\beta_z^\top z_{si}]\right) + E_q[\sigma_{ws}^{-2}]E_q[b_s]\left(w_{si} - E_q[a_s]\right) + E_q[\sigma_x^{-2}]E_q(\mu_x).$$

Recognizing Equation (S.A.21) as the log-density of a univariate normal, we conclude

$$q(x_{si}) = \mathcal{N}\left(m_{x,si}, v_{x,si}\right), \qquad (S.A.22)$$

with

$$v_{x,si} = \left(\omega_{si}E_q[\beta_x^2] + E_q[\sigma_{ws}^{-2}]E_q[b_s^2] + E_q[\sigma_x^{-2}]\right)^{-1},$$

$$m_{x,si} = v_{x,si}\begin{pmatrix} \omega_{si}E_q[\beta_x]\left(z_{si}^* - E_q[\beta_{0s}] - E_q[\beta_z^\top z_{si}]\right) \\ +E_q[\sigma_{ws}^{-2}]E_q[b_s]\left(w_{si} - E_q[a_s]\right) + E_q[\sigma_x^{-2}]E_q(\mu_x) \end{pmatrix},$$

which is equivalent to Equation (20) in the main text.

S.A.5 Variational posterior for $(\boldsymbol{\mu_x}, \boldsymbol{\sigma_x^2})$

Finally, we derive the updates for the hyperparameters governing the marginal distribution of the biomarker x_{si}. The prior is Normal–Inverse-Gamma, the corresponding log-likelihood contributions are,

$$\log p(\sigma_x^2) = -(\alpha_x^0 + 1)\log\sigma_x^2 - \frac{\gamma_x^0}{\sigma_x^2} + \text{const}, \qquad (S.A.23)$$

$$\log p(\mu_x|\sigma_x^2) = -\frac{1}{2}\log\sigma_x^2 - \frac{\kappa_x^0}{2\sigma_x^2}(\mu_x - m_x^0)^2 + \text{const.} \quad (S.A.24)$$

Collect all latent and observed x_{si} into $\{x_{si}\}_{s,i}$, and denote $N = \sum_s n_s$. Conditional on (μ_x, σ_x^2), the likelihood is

$$\log p(x|\mu_x, \sigma_x^2) = -\frac{N}{2}\log(\sigma_x^2) - \frac{1}{2\sigma_x^2}\sum_{s,i}(x_{si} - \mu_x)^2 + \text{const.} \quad (S.A.25)$$

The generic update for (μ_x, σ_x^2) is

$$\log q^*(\mu_x, \sigma_x^2) = E_{q(x)}\left[\sum_{s,i}\log p(x|\mu_x, \sigma_x^2) + \log p(\mu_x|\sigma_x^2) + \log p(\sigma_x^2)\right] + \text{const.} \quad (S.A.26)$$

Define the standard sufficient statistics

$$\bar{x} = \frac{1}{N}\sum_{s,i} E_q[x_{si}], S_{x^2} = \sum_{s,i} E_q[x_{si}^2]. \quad (S.A.27)$$

Completing the square in μ_x gives

$$\log q^*(\mu_x|\sigma_x^2) \propto -\frac{N + \kappa_x^0}{2\sigma_x^2}\left(\mu_x - \frac{N\bar{x} + \kappa_x^0 m_x^0}{N + \kappa_x^0}\right)^2. \quad (S.A.28)$$

Collecting the remaining terms in σ_x^2, we obtain

$$\log q^*(\sigma_x^2) = -\left(\frac{N}{2} + \alpha_x^0 + 1\right)\log\sigma_x^2 - \frac{\frac{1}{2}\left[(S_{x^2} - N\bar{x}^2) + \frac{N\kappa_x^0(\bar{x} - m_x^0)^2}{N + \kappa_x^0}\right] + \gamma_x^0}{\sigma_x^2} + \text{const.} \quad (S.A.29)$$

So, the Normal–Inverse-Gamma update are

$$q(\sigma_x^2) = \text{InvGamma}(\alpha_x, \gamma_x), \quad (S.A.30)$$

and

$$q(\mu_x|\sigma_x^2) = \mathcal{N}\left(m_x, \frac{\sigma_x^2}{\kappa_x}\right), \tag{S.A.31}$$

with

$$\alpha_x = \frac{N}{2} + \alpha_x^0, \gamma_x = \frac{1}{2}\left[(S_{x^2} - N\bar{x}^2) + \frac{N\kappa_x^0(\bar{x} - m_x^0)^2}{N + \kappa_x^0}\right] + \gamma_x^0,$$

$$m_x = \frac{N\bar{x} + \kappa_x^0 m_x^0}{N + \kappa_x^0}, \kappa_x = N + \kappa_x^0,$$

which reproduces the updates in Equations (21)-(23) of the main text.

S.B Variational Gamma approximation for the Weibull shape parameter

For the Weibull outcome model in Section 2.3, the ELBO contribution involving the shape parameter ρ arises from the survival likelihood and the prior on ρ, Conditional on the current variational distributions for all other parameters, the log-density as a function of ρ can be written as

$$J(\rho) = \sum_{s,i}\{\delta_{si}[\log\rho + (\rho - 1)\log t_{si}] - {t_{si}}^{\rho} E_q[e^{\eta_{si}}]\} + (\alpha_\rho^0 - 1)\log\rho - \gamma_\rho^0\rho. \tag{S.B.1}$$

where $E_q[e^{\eta_{si}}]$ is taken with respect to the current variational distribution of the linear predictor η_{si}, and α_ρ^0, γ_ρ^0 are the hyperparameters of the Gamma prior $\rho \sim Gamma(\alpha_\rho^0, \gamma_\rho^0)$. Terms not depending on ρ are omitted.

We consider a variational family for $q(\rho)$ given by a Gamma distribution with shape $\alpha_\rho > 0$ and rate $\gamma_\rho > 0$,

$$q(\rho) = \text{Gamma}(\rho|\alpha_\rho, \gamma_\rho) = \frac{{\gamma_\rho}^{\alpha_\rho}}{\Gamma(\alpha_\rho)}\rho^{\alpha_\rho - 1}\exp(-\gamma_\rho\rho). \tag{S.B.2}$$

Under this variational distribution, the following expectations are standard,

$$E_q[\rho] = \frac{\alpha_\rho}{\gamma_\rho}, E_q[\log\rho] = \psi(\alpha_\rho) - \log\gamma_\rho, \qquad (S.B.3)$$

where $\psi(\cdot)$ is the digamma function, In addition, for each observed time $t_{si} > 0$,

$$E_q({t_{si}}^{\rho}) = E_q[\exp(\rho\log t_{si})] = \left(1 - \frac{\log t_{si}}{\gamma_\rho}\right)^{-\alpha_\rho}, \gamma_\rho > \max_{s,i}\{\log t_{si}\} \qquad (S.B.4)$$

which follows from the moment generating function of a Gamma (shape α_ρ, rate γ_ρ) distribution.

Let

$$A = \sum_{s,i}\delta_{si}, U = \sum_{s,i}\delta_{si}\log t_{si}, C_{si} = E_q(e^{\eta_{si}}), M_{si} = E({t_{si}}^{\rho}) = \left(1 - \frac{\log t_{si}}{\gamma_\rho}\right)^{-\alpha_\rho}. \qquad (S.B.5)$$

Using the mean-field factorization $q(\rho,\eta) = q(\rho)\, q(\eta)$, we have

$$E_q[{t_{si}}^{\rho} e^{\eta_{si}}] = E_q[{t_{si}}^{\rho}]E_q[e^{\eta_{si}}] = C_{si}M_{si}, \qquad (S.B.6)$$

The ELBO contribution involving $q(\rho)$ is

$$\mathcal{L}_\rho(\alpha_\rho, \gamma_\rho) = E_q[J(\rho)] - E_q[\log q(\rho)] + const, \qquad (S.B.7)$$

where $E_q[\cdot]$ is with respect to $q(\rho)$ in Equation (S.B.2), and "const" denotes terms independent of α_ρ and γ_ρ. $E_q[J(\rho)]$ and $E_q[\log q(\rho)]$ can be calculated as,

$$E_q[J(\rho)] = (A + \alpha_\rho^0 - 1)E_q[\log\rho] + (U - \gamma_\rho^0)E_q[\rho] - \sum_{s,i} C_{si}M_{si} \qquad (S.B.8)$$

$$E_q[\log q(\rho)] = \alpha_\rho \log\gamma_\rho - \log\Gamma(\alpha_\rho) + (\alpha_\rho - 1)E_q[\log\rho] - \gamma_\rho E_q[\rho] \qquad (S.B.9)$$

Combining Equations (S.B.3)-(S.B.4) and Equations (S.B.7)-(S.B.9),

$$\mathcal{L}_\rho(\alpha_\rho, \gamma_\rho) = (A + \alpha_\rho^0 - \alpha_\rho)\psi(\alpha_\rho) - (A + \alpha_\rho^0)\log\gamma_\rho + (U - \gamma_\rho^0)\frac{\alpha_\rho}{\gamma_\rho} - \sum_{s,i} C_{si}M_{si} + \alpha_\rho + \log\Gamma(\alpha_\rho) + const\,. \qquad (S.B.10)$$

The gradients of $\mathcal{L}_\rho$ with respect to α_ρ and γ_ρ are

$$\frac{\partial L_\rho}{\partial \alpha_\rho} = (A + \alpha_\rho^0 - \alpha_\rho)\psi(\alpha_\rho)' + \frac{U - \gamma_\rho^0}{\gamma_\rho} + \sum_{s,i} C_{si}M_{si}log\left(1 - \frac{\log t_{si}}{\gamma_\rho}\right) + 1. \qquad (S.B.11)$$

$$\frac{\partial L_\rho}{\partial \gamma_\rho} = \frac{1}{{\gamma_\rho}^2}\left(-(A + \alpha_\rho^0)\gamma_\rho - \alpha_\rho(U - \gamma_\rho^0) + \sum_{s,i}\frac{C_{si}M_{si}\,\alpha_\rho\log t_{si}}{1 - \frac{\log t_{si}}{\gamma_\rho}}\right). \qquad (S.B.12)$$

In principle, one can update α_ρ and γ_ρ by gradient-based maximization of $\mathcal{L}_\rho(\alpha_\rho, \gamma_\rho)$, subject to the constraints $\alpha_\rho > 0$ and $\gamma_\rho > \max_{s,i}\{\log t_{si}\}$ to ensure that the moments in (S.B.4) exist. In our implementation, we enforced these constraints via softplus reparameterizations of the free optimization variables. However, we found that the resulting two-dimensional updates for α_ρ and γ_ρ were numerically less stable and substantially slower than a direct Newton–Raphson update for a single shape parameter ρ (or $\xi = \log\rho$). For this reason, the main algorithm presented in the paper uses a Newton–Raphson update for the Weibull shape parameter rather than a Gamma variational family for $q(\rho)$.

S.C Implementation details

Defaults settings.

All simulations and the real-data analysis were implemented in Python using the CAVI algorithms summarized in Algorithms 1-2. Unless otherwise noted, the algorithm was run with default settings $t_{max} = 1000$, $\epsilon_1 = 10^{-3}$, and $\epsilon_{\text{tol}} = 10^{-4}$, with random seed set to 0 for reproducibility. The stopping rule follows the parameter-change criterion in Algorithms 1-2, that is, at iteration t, we stack all free variational parameters into a single vector $\theta^{(t)}$ and compute a relative change metric

$$\text{diff} = \max\left(\frac{\left|\theta^{(t)} - \theta^{(t-1)}\right|}{\left|\theta^{(t-1)}\right| + \epsilon_1}\right)$$

where the absolute value, division, and max are taken element-wise. The small constant ϵ_1 acts as a numerical stabilizer to avoid division by values close to zero. The algorithm terminates when $\text{diff} \leq \epsilon_{\text{tol}}$ or when t reaches t_{max}. In addition, confidence intervals are obtained by nonparametric bootstrap, and we use $B = 200$ bootstrap samples by default with the same convergence settings as above.

Initialization.

We initialized the variational parameters using a combination of prior-based and data-driven starting values. Regression coefficients were initialized at the prior mean m_β^0, with prior covariance Σ_β^0, where $m_\beta^0 = 0$ and $\Sigma_\beta^0 = I$ by default. For each study s, the calibration parameters $u_s = (a_s, b_s)^\top$ were initialized using an OLS fit of w on observed reference measurements x^o within that study when such paired data were available. If a study had no reference measurements, we initialized u_s at the prior mean $m_{ab}^0 = 0$ with prior covariance $\Sigma_{ab}^0 = I_2$. The study-specific measurement error variances σ_{ws}^2 were initialized from the OLS residual variance when available and set to 1.0 otherwise. The corresponding inverse-gamma variational parameters were initialized as $\alpha_w = \alpha_w^0 + 0.5n_s$ and $\gamma_w = \gamma_w^0 + 0.5n_s\hat{\sigma}_{ws}^2$, with $\alpha_w^0 = \gamma_w^0 = 1$. For the latent reference-scale biomarker distribution, if the number of observed reference measurements was small, we initialized m_x at the prior mean $m_x^0 = 0$ and set $\sigma_x^2 = 1$; otherwise, we used the empirical mean and variance from x^o. The corresponding Normal–Inverse-Gamma variational parameters were initialized with $\kappa_x = \kappa_x^0 + \sum_s n_s$, $\alpha_x = \alpha_x^0 + (\sum_s n_s)/2$, and $\gamma_x = \gamma_x^0 + 0.5(\sum_s n_s)\hat{\sigma}_x^2$, where $\kappa_x^0 = \alpha_x^0 = \gamma_x^0 = 1$ by default. Missing reference biomarker values x_{si} were initialized as $q(x_{si}) = \mathcal{N}(m_x, \sigma_x^2)$. For Weibull models, initialization depends on the chosen specification for the shape parameter ρ. Under the gamma prior, ρ is initialized at the prior mean $\rho^{(0)} = \alpha_\rho^0/\gamma_\rho^0$. Under the lognormal prior, ρ is initialized at $\rho^{(0)} = \exp\left(m_\xi^0 + 0.5\left(\sigma_\xi^2\right)^0\right)$, where $\xi = \log\rho$.

S.D Comparison of operating characteristics under multiple scenarios

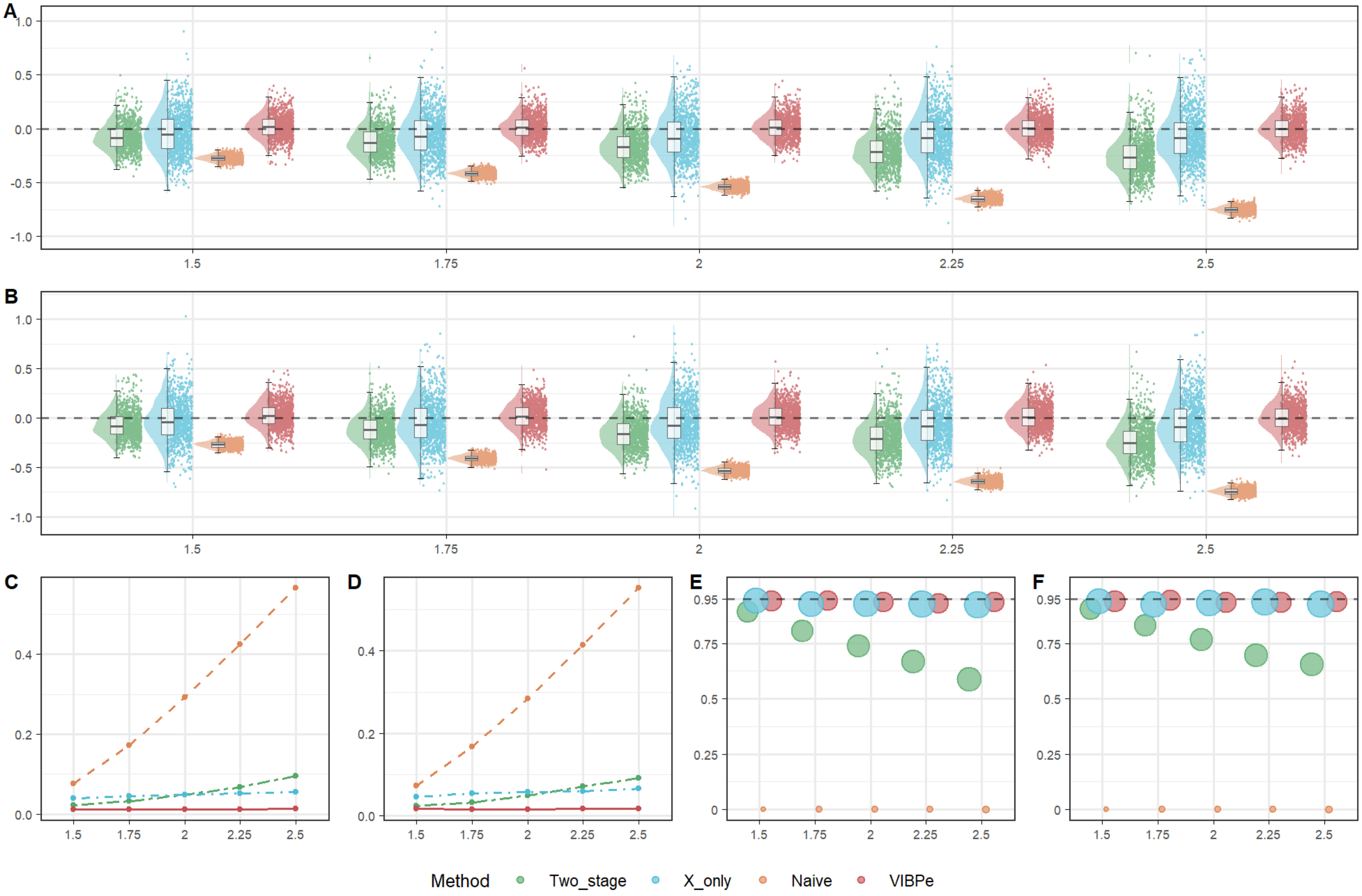

Figure S1. Comparison of operating characteristics under Scenario 1 (exponential baseline scenario) with per-study sample size $n_s = 50$ for the proposed VIBPe, naive, x-only, and two-stage methods. A. Bias under censoring rate 0.1; B. Bias under censoring rate 0.3; C. MSE under censoring rate 0.1; D. MSE under censoring rate 0.3; E. Coverage rate under censoring rate 0.1; F. Coverage rate under censoring rate 0.3. The true effect is indexed on the HR scale ($HR = \exp(\beta)$) for presentation, whereas Bias, MSE, and coverage are computed for β on the log-HR scale.

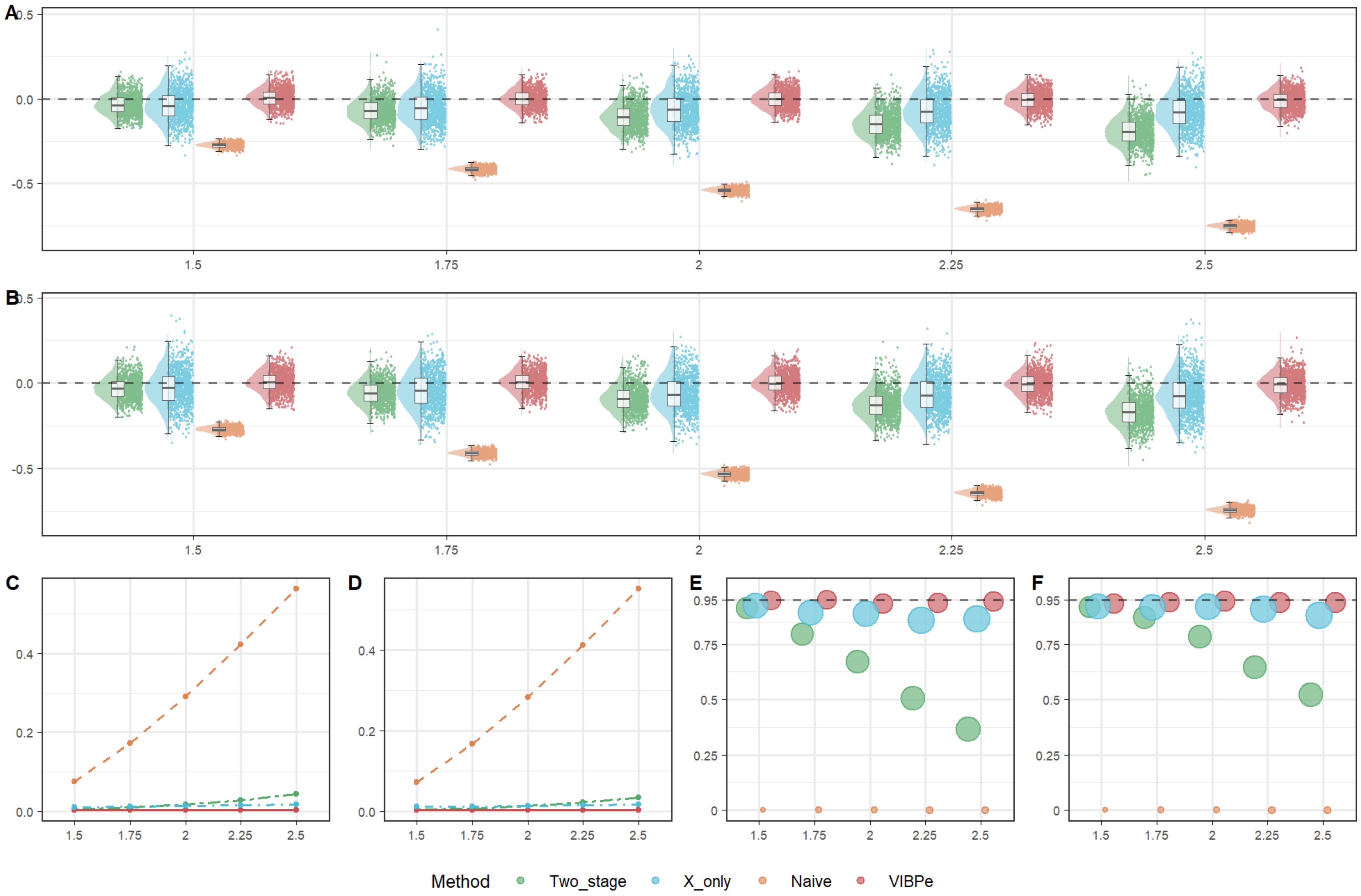

Figure S2. Comparison of operating characteristics under Scenario 1 (exponential baseline scenario) with per-study sample size $n_s = 200$ for the proposed VIBPe, naive, x-only, and two-stage methods. A. Bias under censoring rate 0.1; B. Bias under censoring rate 0.3; C. MSE under censoring rate 0.1; D. MSE under censoring rate 0.3; E. Coverage rate under censoring rate 0.1; F. Coverage rate under censoring rate 0.3. The true effect is indexed on the HR scale ($HR = \exp(\beta)$) for presentation, whereas Bias, MSE, and coverage are computed for β on the log-HR scale.

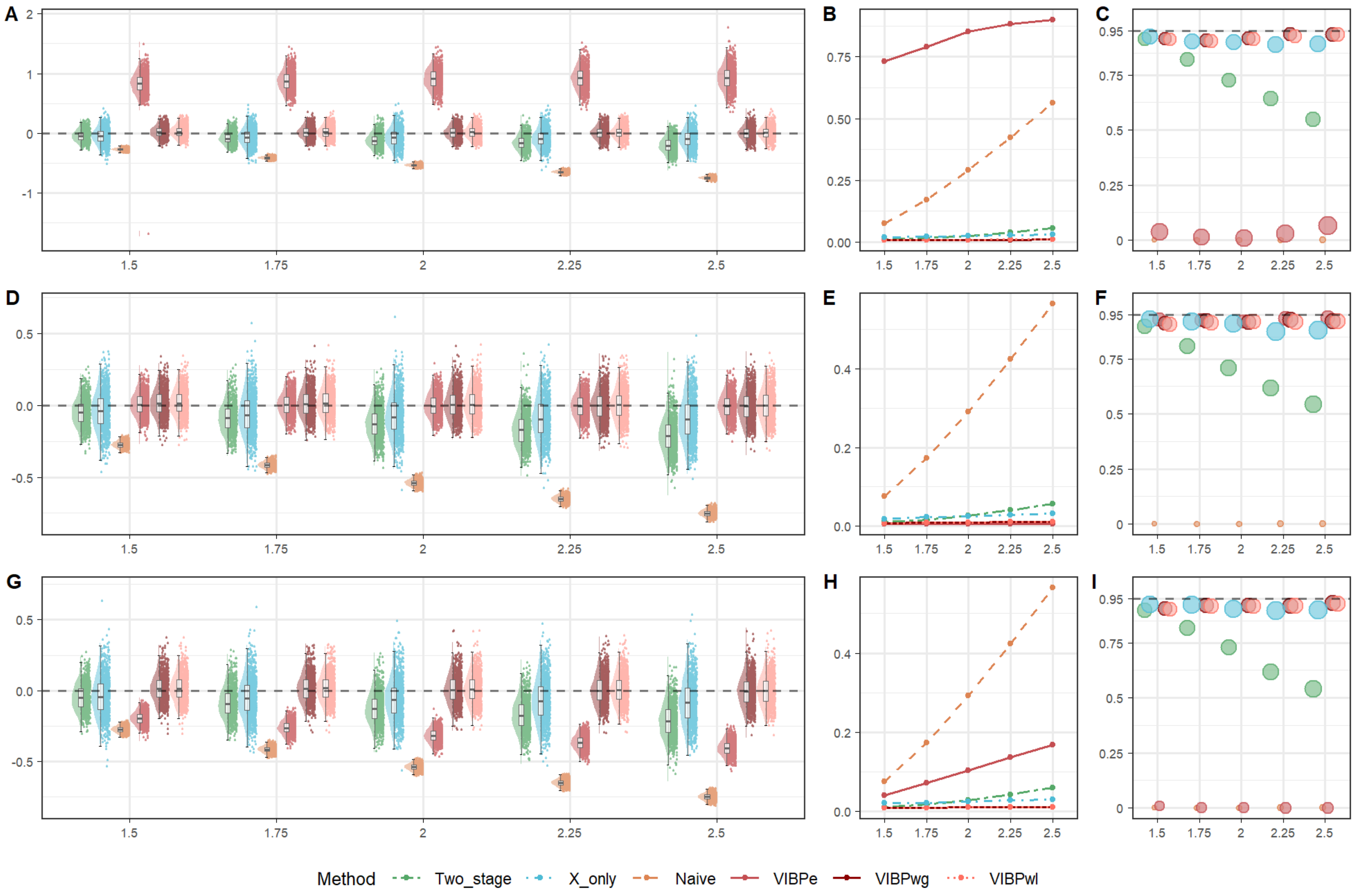

Figure S3. Comparison of operating characteristics under scenario 6 (Weibull baseline scenario) with censoring rate 0.1 for the VIBPe, VIBPwg, VIBPwl, naive, x-only, and two-stage methods. A. Bias under ρ=0.5; B. MSE under ρ=0.5; C. Coverage rate under ρ=0.5; D. Bias under ρ=1.0; E. MSE under ρ=1.0; F. Coverage rate under ρ=1.0; G. Bias under ρ=1.5; H. MSE under ρ=1.5; I. Coverage rate under ρ=1.5. The true effect is indexed on the HR scale ($HR = \exp(\beta)$) for presentation, whereas Bias, MSE, and coverage are computed for β on the log-HR scale.

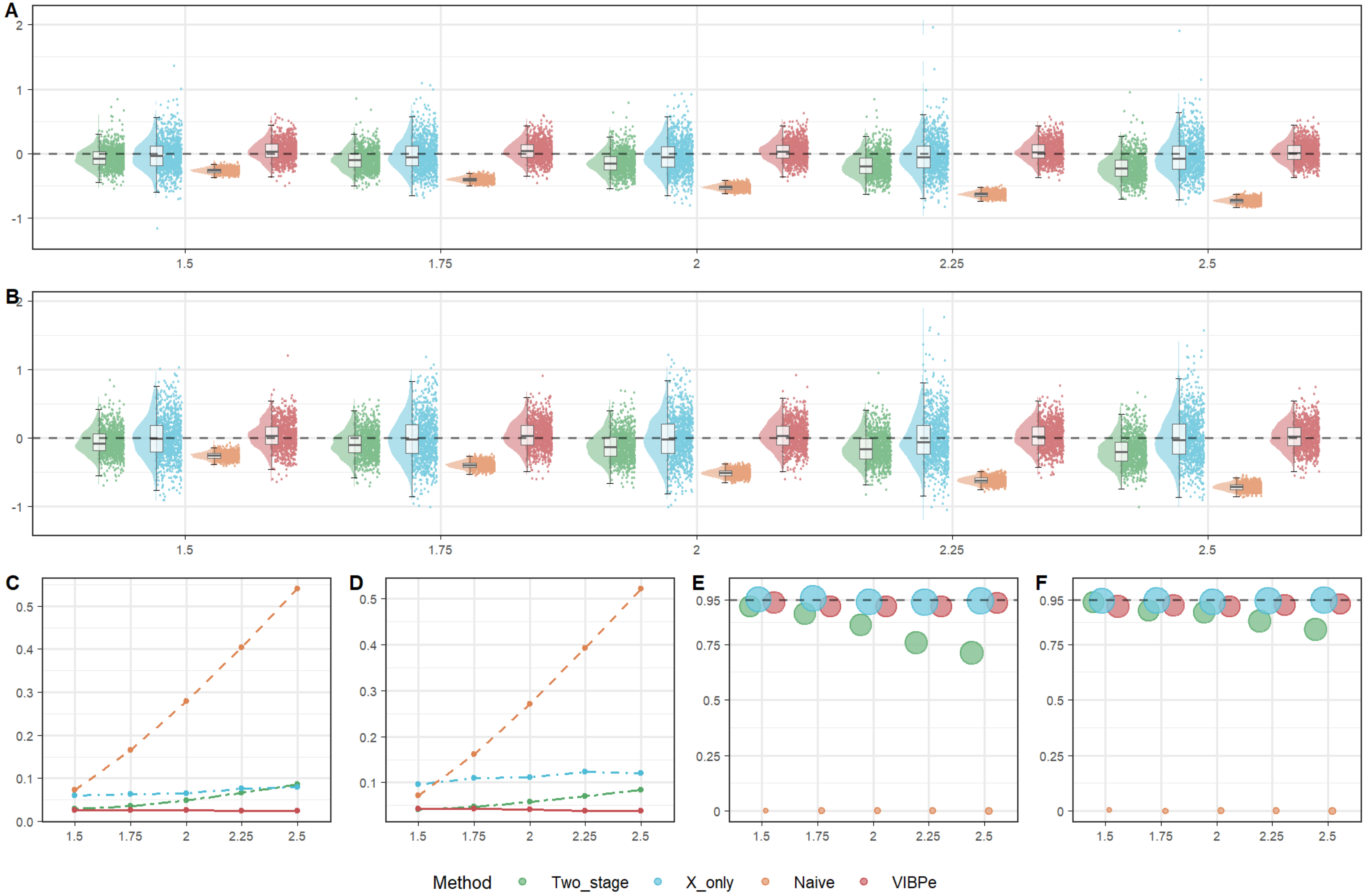

Figure S4. Comparison of operating characteristics under Scenario 2 (exponential high censoring scenario) with per-study sample size $n_s = 50$ for the proposed VIBPe, naive, x-only, and two-stage methods. A. Bias under censoring rate 0.5; B. Bias under censoring rate 0.7; C. MSE under censoring rate 0.5; D. MSE under censoring rate 0.7; E. Coverage rate under censoring rate 0.5; F. Coverage rate under censoring rate 0.7. The true effect is indexed on the HR scale ($HR = \exp(\beta)$) for presentation, whereas Bias, MSE, and coverage are computed for β on the log-HR scale.

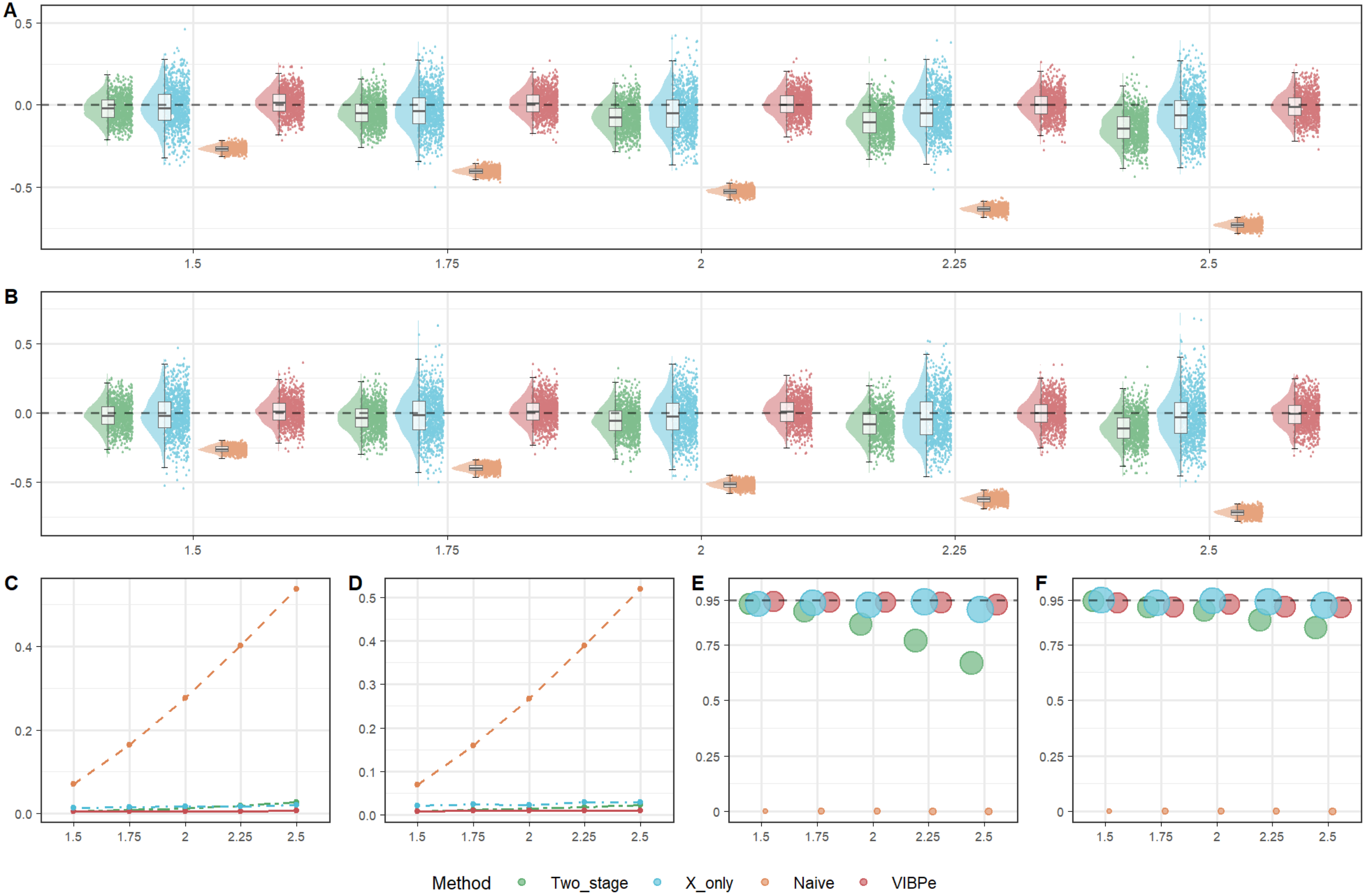

Figure S5. Comparison of operating characteristics under Scenario 2 (exponential high censoring scenario) with per-study sample size $n_s = 200$ for the proposed VIBPe, naive, x-only, and two-stage methods. A. Bias under censoring rate 0.5; B. Bias under censoring rate 0.7; C. MSE under censoring rate 0.5; D. MSE under censoring rate 0.7; E. Coverage rate under censoring rate 0.5; F. Coverage rate under censoring rate 0.7. The true effect is indexed on the HR scale ($HR = \exp(\beta)$) for presentation, whereas Bias, MSE, and coverage are computed for β on the log-HR scale.

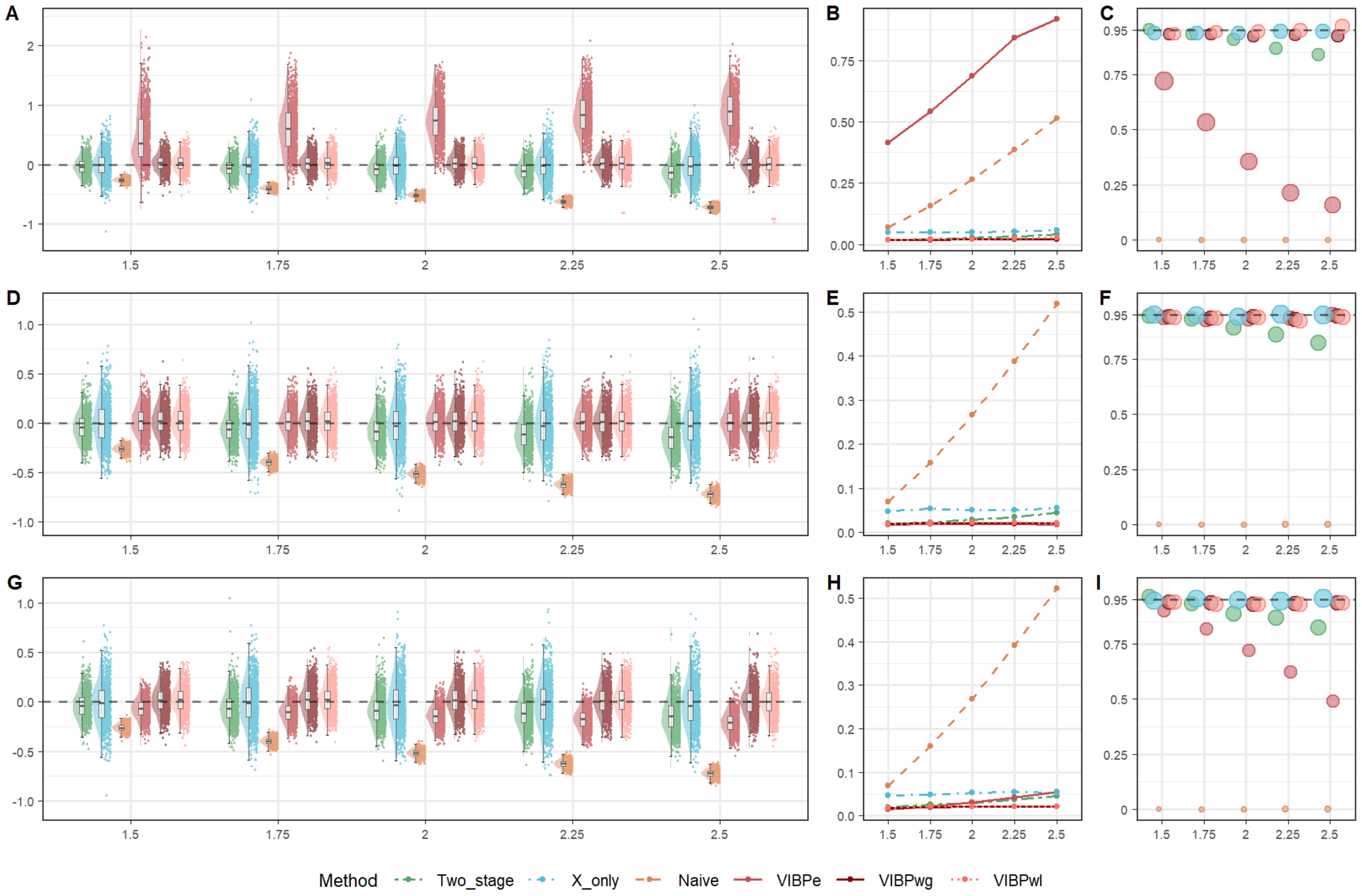

Figure S6. Comparison of operating characteristics under scenario 7 (Weibull high censoring scenario) with censoring rate 0.7 for the VIBPe, VIBPwg, VIBPwl, naive, x-only, and two-stage methods. A. Bias under ρ=0.5; B. MSE under ρ=0.5; C. Coverage rate under ρ=0.5; D. Bias under ρ=1.0; E. MSE under ρ=1.0; F. Coverage rate under ρ=1.0; G. Bias under ρ=1.5; H. MSE under ρ=1.5; I. Coverage rate under ρ=1.5. The true effect is indexed on the HR scale ($HR = \exp(\beta)$) for presentation, whereas Bias, MSE, and coverage are computed for β on the log-HR scale.

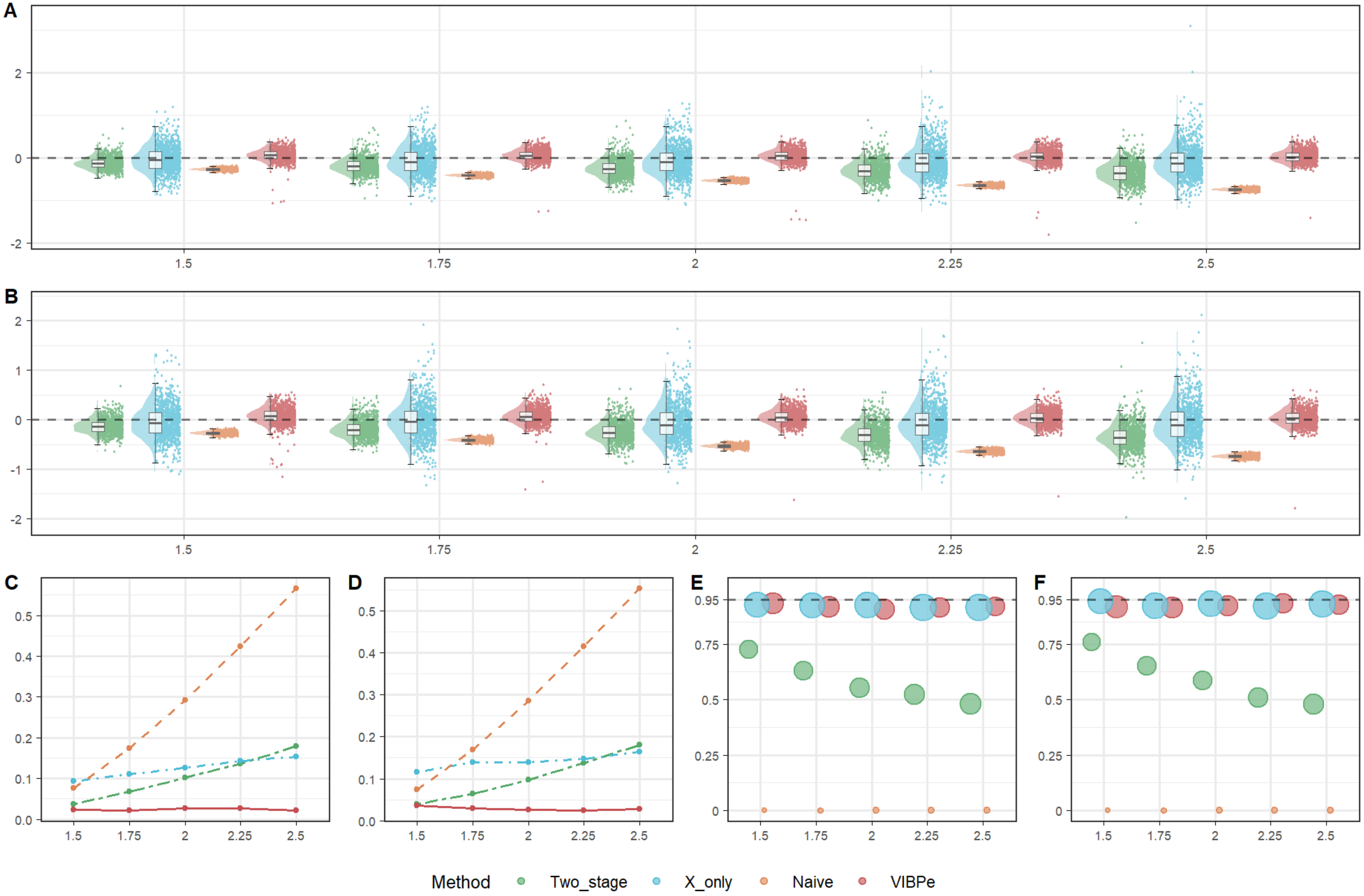

Figure S7. Comparison of operating characteristics under Scenario 3 (exponential sparser calibration subset scenario) with per-study sample size $n_s = 50$ for the proposed VIBPe, naive, x-only, and two-stage methods. A. Bias under censoring rate 0.1; B. Bias under censoring rate 0.3; C. MSE under censoring rate 0.1; D. MSE under censoring rate 0.3; E. Coverage rate under censoring rate 0.1; F. Coverage rate under censoring rate 0.3. The true effect is indexed on the HR scale ($HR = \exp(\beta)$) for presentation, whereas Bias, MSE, and coverage are computed for β on the log-HR scale.

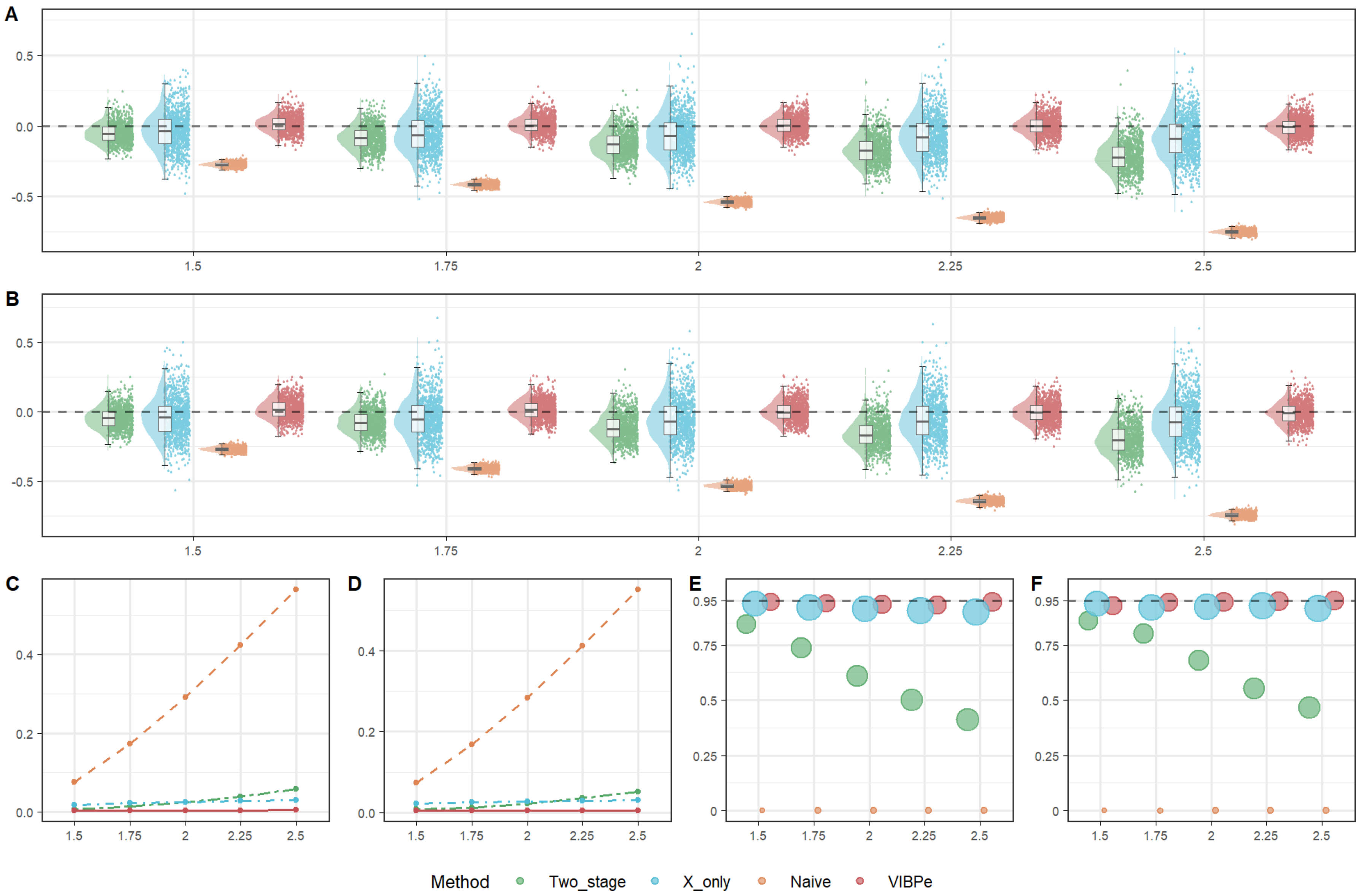

Figure S8. Comparison of operating characteristics under Scenario 3 (exponential sparser calibration subset scenario) with per-study sample size $n_s = 200$ for the proposed VIBPe, naive, x-only, and two-stage methods. A. Bias under censoring rate 0.1; B. Bias under censoring rate 0.3; C. MSE under censoring rate 0.1; D. MSE under censoring rate 0.3; E. Coverage rate under censoring rate 0.1; F. Coverage rate under censoring rate 0.3. The true effect is indexed on the HR scale ($HR = \exp(\beta)$) for presentation, whereas Bias, MSE, and coverage are computed for β on the log-HR scale.

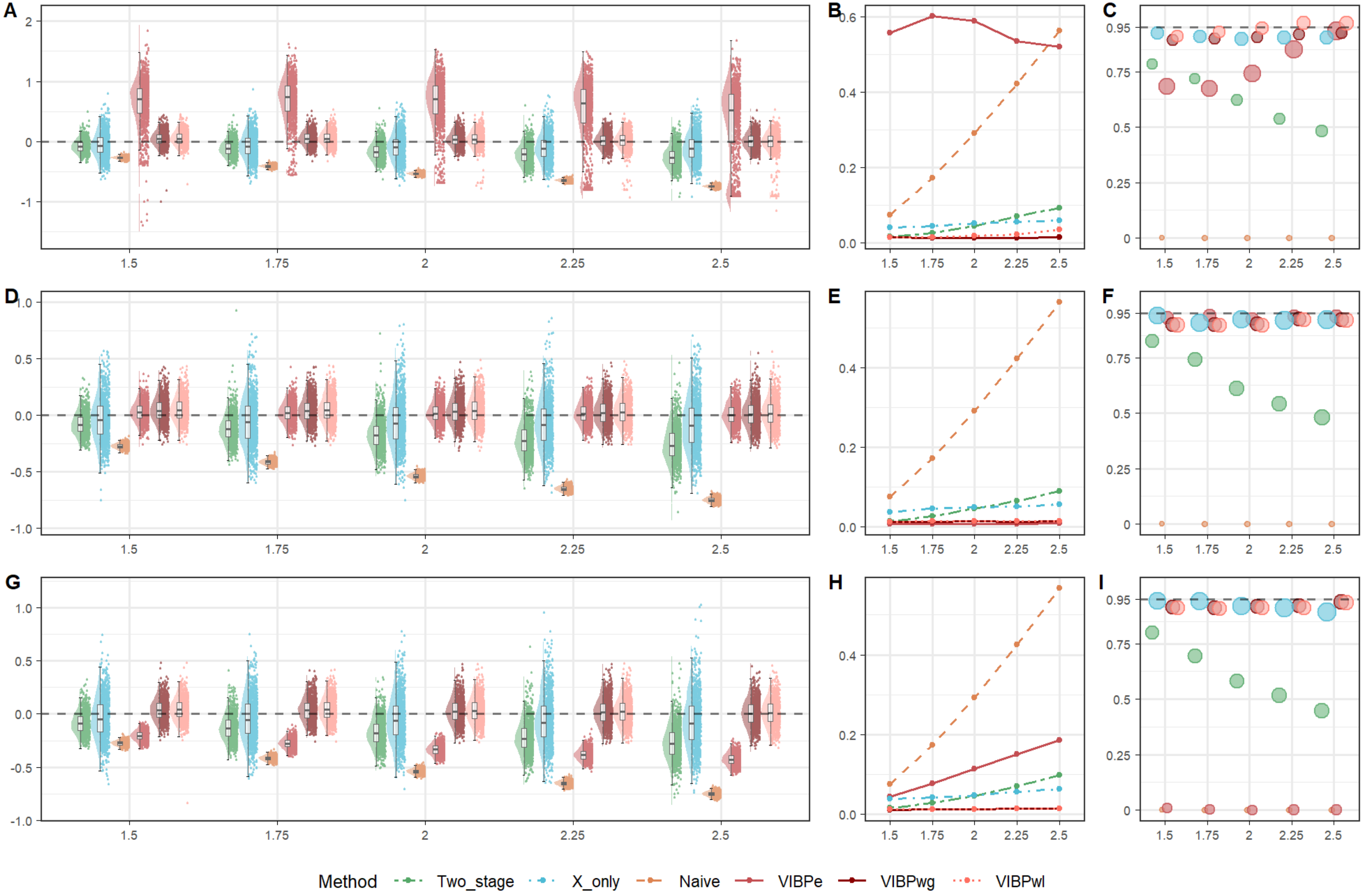

Figure S9. Comparison of operating characteristics under scenario 8 (Weibull sparser calibration subset scenario) with censoring rate 0.1 for the VIBPe, VIBPwg, VIBPwl, naive, x-only, and two-stage methods. A. Bias under ρ=0.5; B. MSE under ρ=0.5; C. Coverage rate under ρ=0.5; D. Bias under ρ=1.0; E. MSE under ρ=1.0; F. Coverage rate under ρ=1.0; G. Bias under ρ=1.5; H. MSE under ρ=1.5; I. Coverage rate under ρ=1.5. The true effect is indexed on the HR scale ($HR = \exp(\beta)$) for presentation, whereas Bias, MSE, and coverage are computed for β on the log-HR scale.

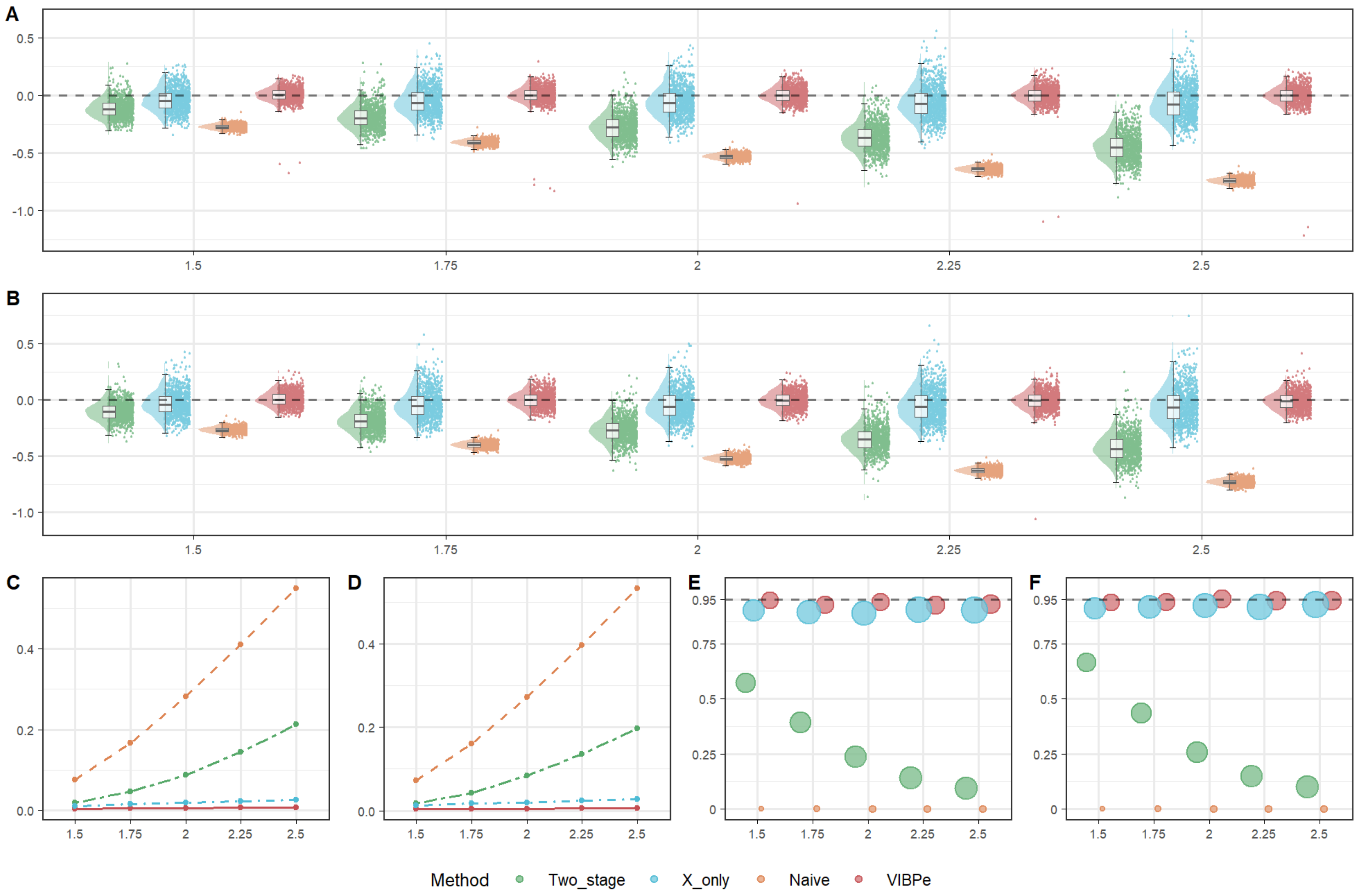

Figure S10. Comparison of operating characteristics under Scenario 4 (exponential high scale scenario) with per-study sample size $n_s = 50$ for the proposed VIBPe, naive, x-only, and two-stage methods. A. Bias under censoring rate 0.1; B. Bias under censoring rate 0.3; C. MSE under censoring rate 0.1; D. MSE under censoring rate 0.3; E. Coverage rate under censoring rate 0.1; F. Coverage rate under censoring rate 0.3. The true effect is indexed on the HR scale ($HR = \exp(\beta)$) for presentation, whereas Bias, MSE, and coverage are computed for β on the log-HR scale.

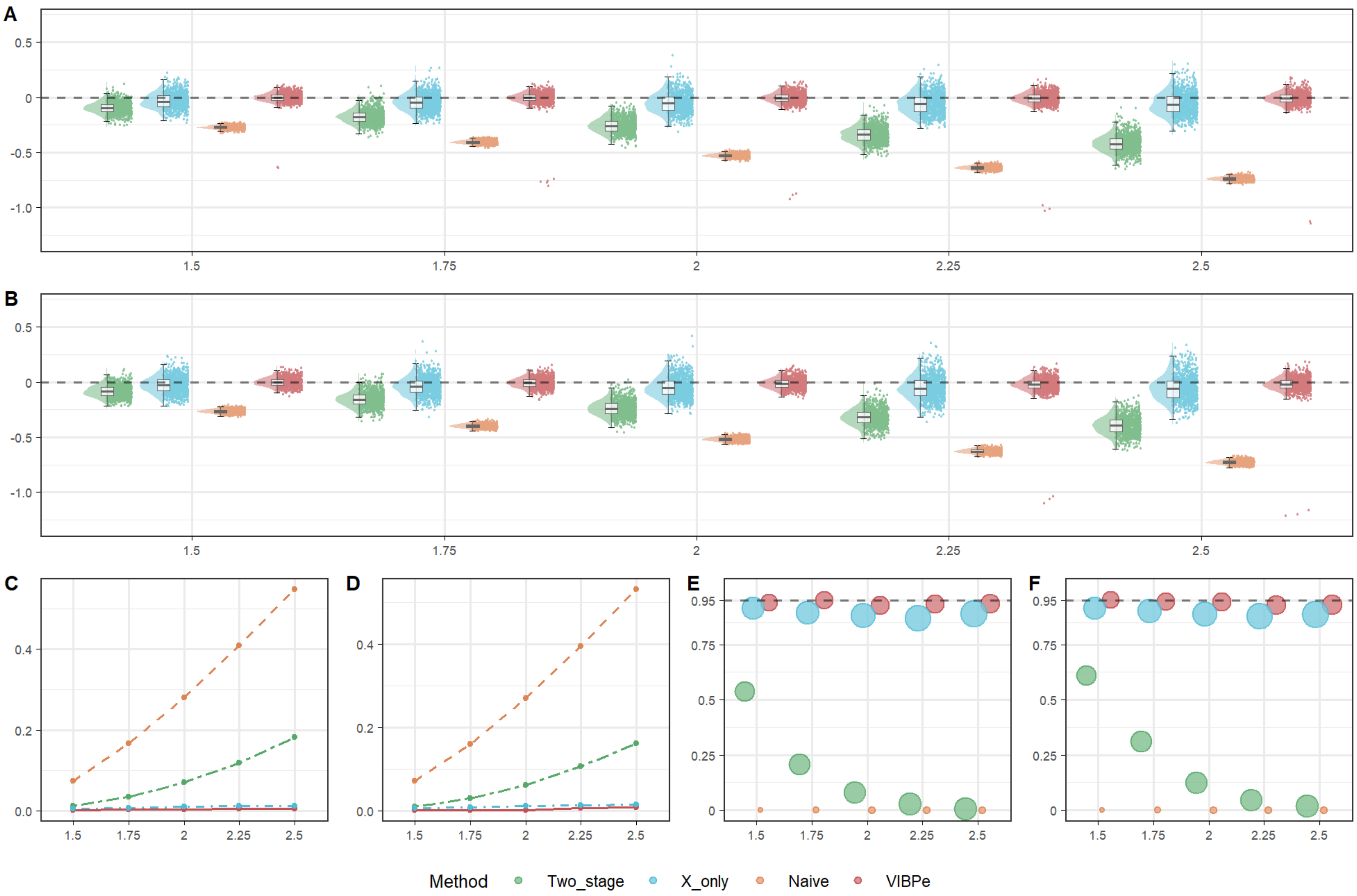

Figure S11. Comparison of operating characteristics under Scenario 4 (exponential high scale scenario) with per-study sample size $n_s = 100$ for the proposed VIBPe, naive, x-only, and two-stage methods. A. Bias under censoring rate 0.1; B. Bias under censoring rate 0.3; C. MSE under censoring rate 0.1; D. MSE under censoring rate 0.3; E. Coverage rate under censoring rate 0.1; F. Coverage rate under censoring rate 0.3. The true effect is indexed on the HR scale ($HR = \exp(\beta)$) for presentation, whereas Bias, MSE, and coverage are computed for β on the log-HR scale.

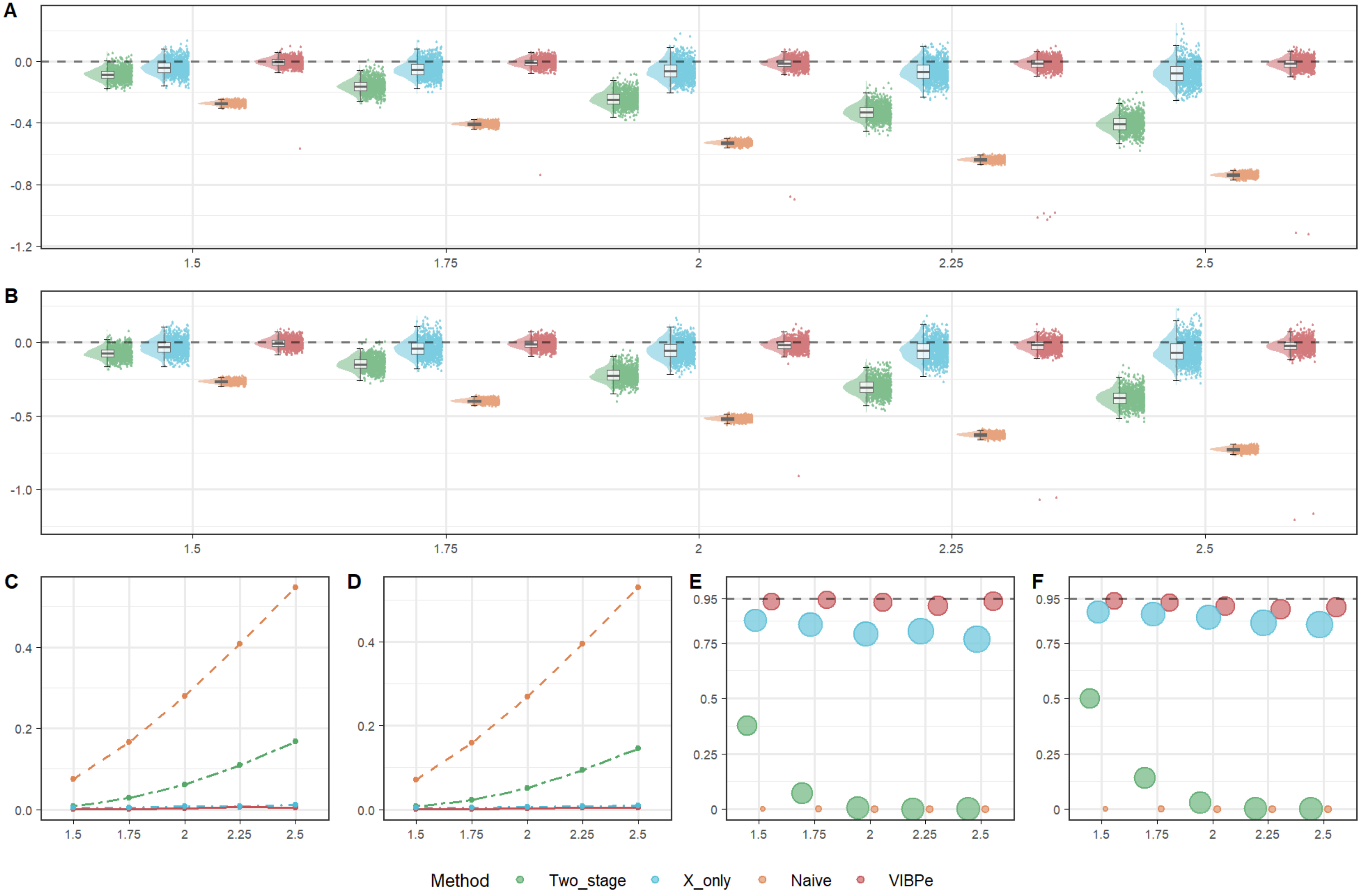

Figure S12. Comparison of operating characteristics under Scenario 4 (exponential high scale scenario) with per-study sample size $n_s = 200$ for the proposed VIBPe, naive, x-only, and two-stage methods. A. Bias under censoring rate 0.1; B. Bias under censoring rate 0.3; C. MSE under censoring rate 0.1; D. MSE under censoring rate 0.3; E. Coverage rate under censoring rate 0.1; F. Coverage rate under censoring rate 0.3. The true effect is indexed on the HR scale ($HR = \exp(\beta)$) for presentation, whereas Bias, MSE, and coverage are computed for β on the log-HR scale.

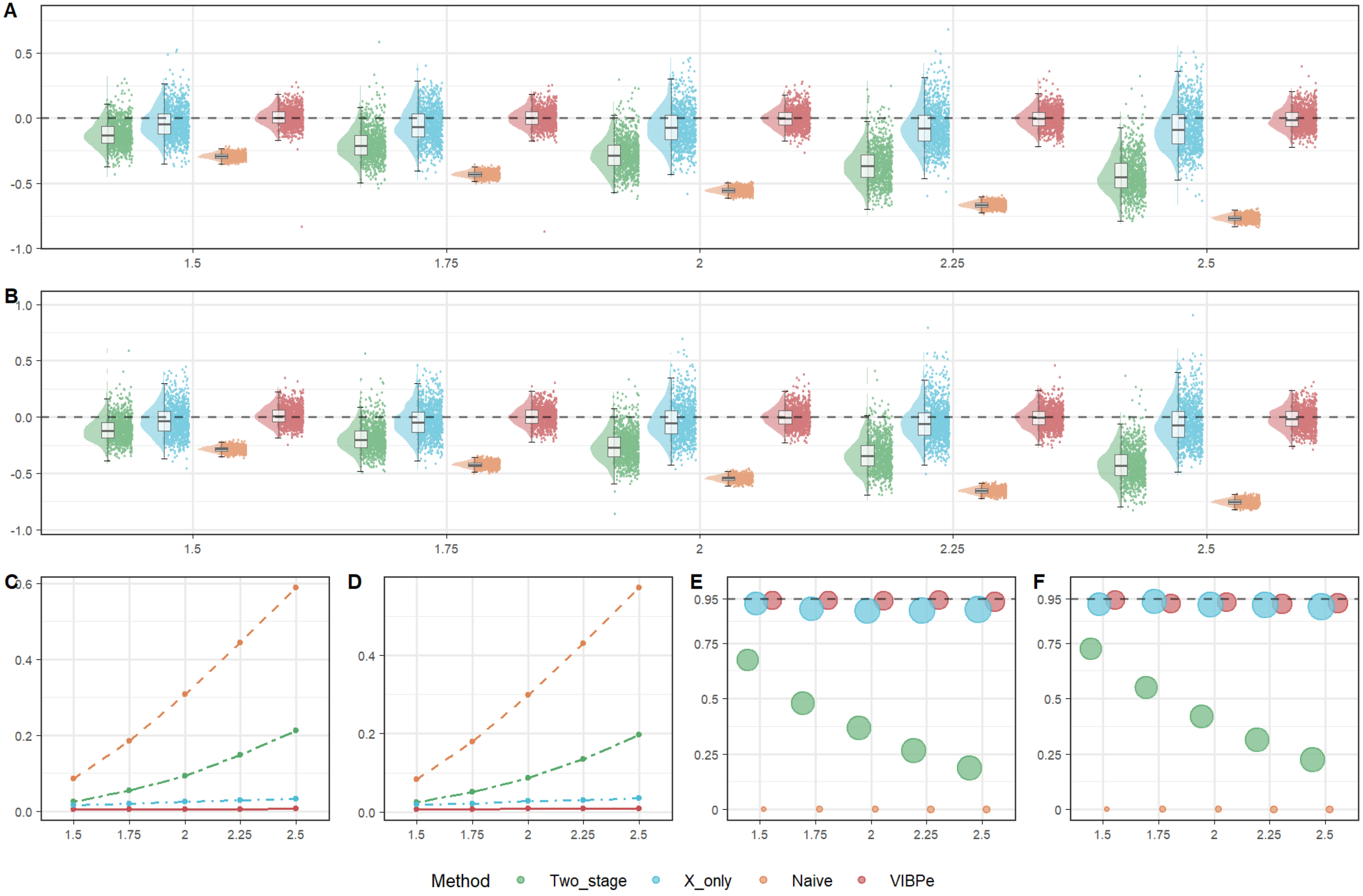

Figure S13. Comparison of operating characteristics under Scenario 5 (exponential high noise scenario) with per-study sample size $n_s = 50$ for the proposed VIBPe, naive, x-only, and two-stage methods. A. Bias under censoring rate 0.1; B. Bias under censoring rate 0.3; C. MSE under censoring rate 0.1; D. MSE under censoring rate 0.3; E. Coverage rate under censoring rate 0.1; F. Coverage rate under censoring rate 0.3. The true effect is indexed on the HR scale ($HR = \exp(\beta)$) for presentation, whereas Bias, MSE, and coverage are computed for β on the log-HR scale.

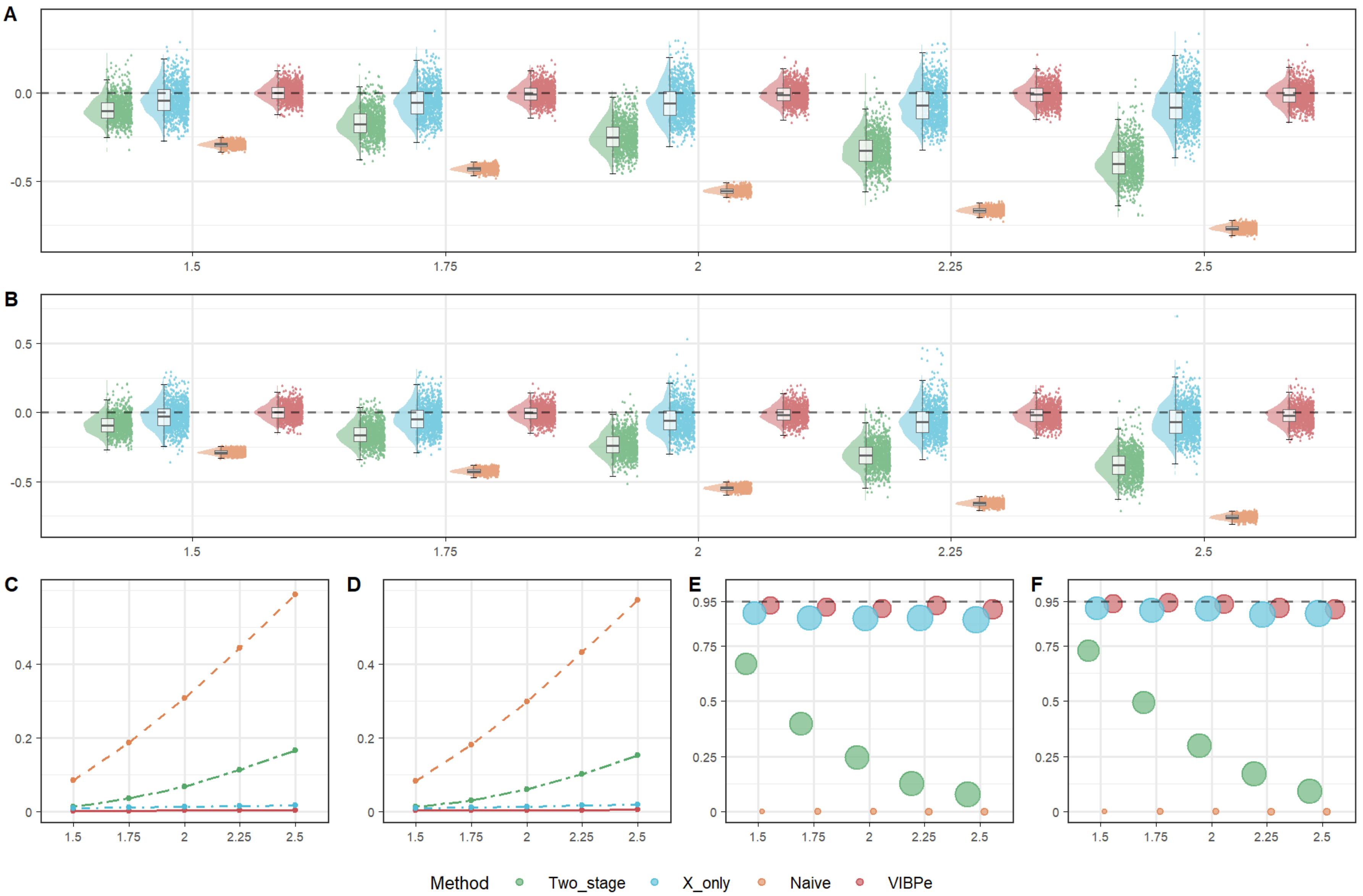

Figure S14. Comparison of operating characteristics under Scenario 5 (exponential high noise scenario) with per-study sample size $n_s = 100$ for the proposed VIBPe, naive, x-only, and two-stage methods. A. Bias under censoring rate 0.1; B. Bias under censoring rate 0.3; C. MSE under censoring rate 0.1; D. MSE under censoring rate 0.3; E. Coverage rate under censoring rate 0.1; F. Coverage rate under censoring rate 0.3. The true effect is indexed on the HR scale ($HR = \exp(\beta)$) for presentation, whereas Bias, MSE, and coverage are computed for β on the log-HR scale.

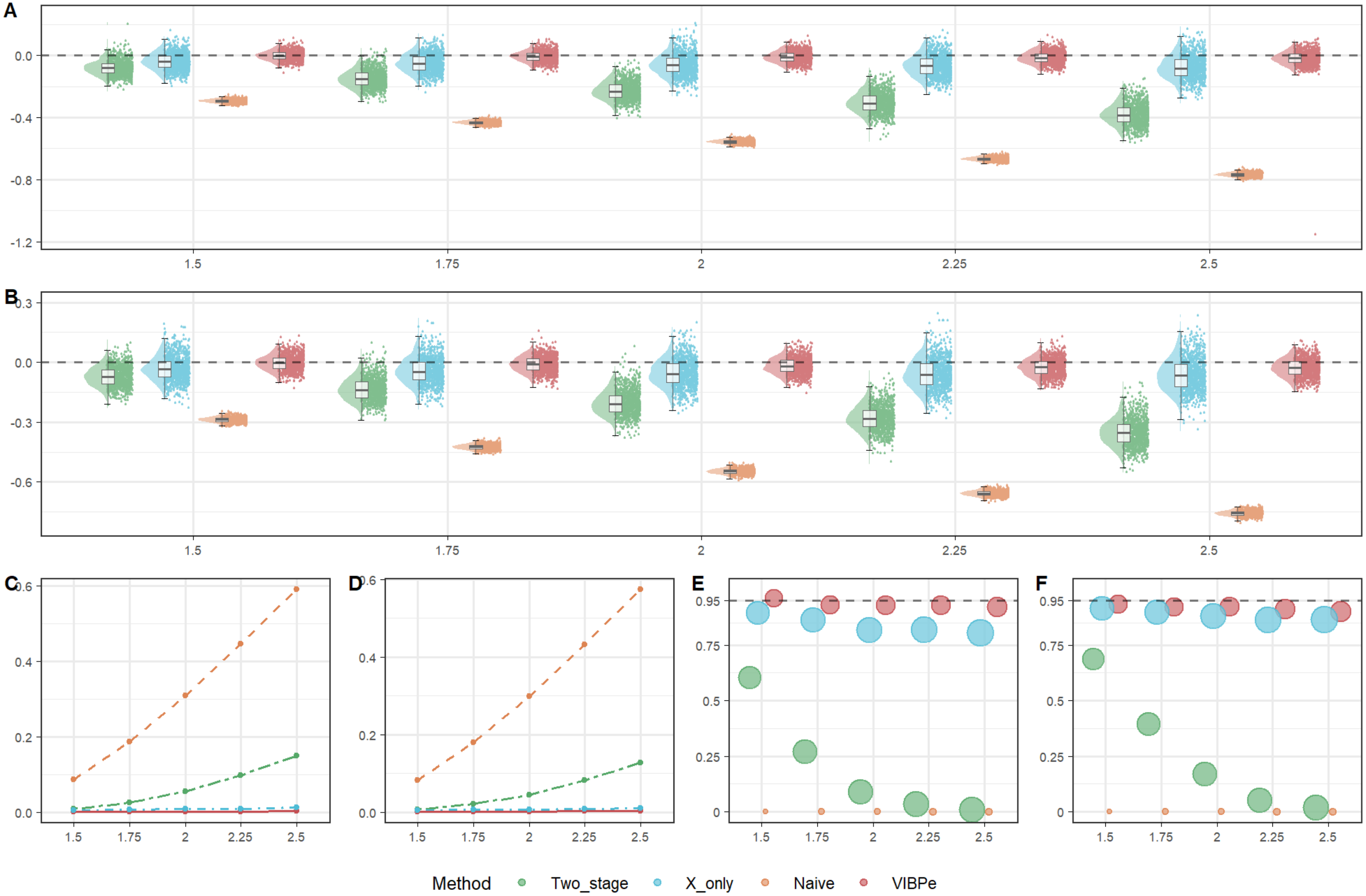

Figure S15. Comparison of operating characteristics under Scenario 5 (exponential high noise scenario) with per-study sample size $n_s = 200$ for the proposed VIBPe, naive, x-only, and two-stage methods. A. Bias under censoring rate 0.1; B. Bias under censoring rate 0.3; C. MSE under censoring rate 0.1; D. MSE under censoring rate 0.3; E. Coverage rate under censoring rate 0.1; F. Coverage rate under censoring rate 0.3. The true effect is indexed on the HR scale ($HR = \exp(\beta)$) for presentation, whereas Bias, MSE, and coverage are computed for β on the log-HR scale.

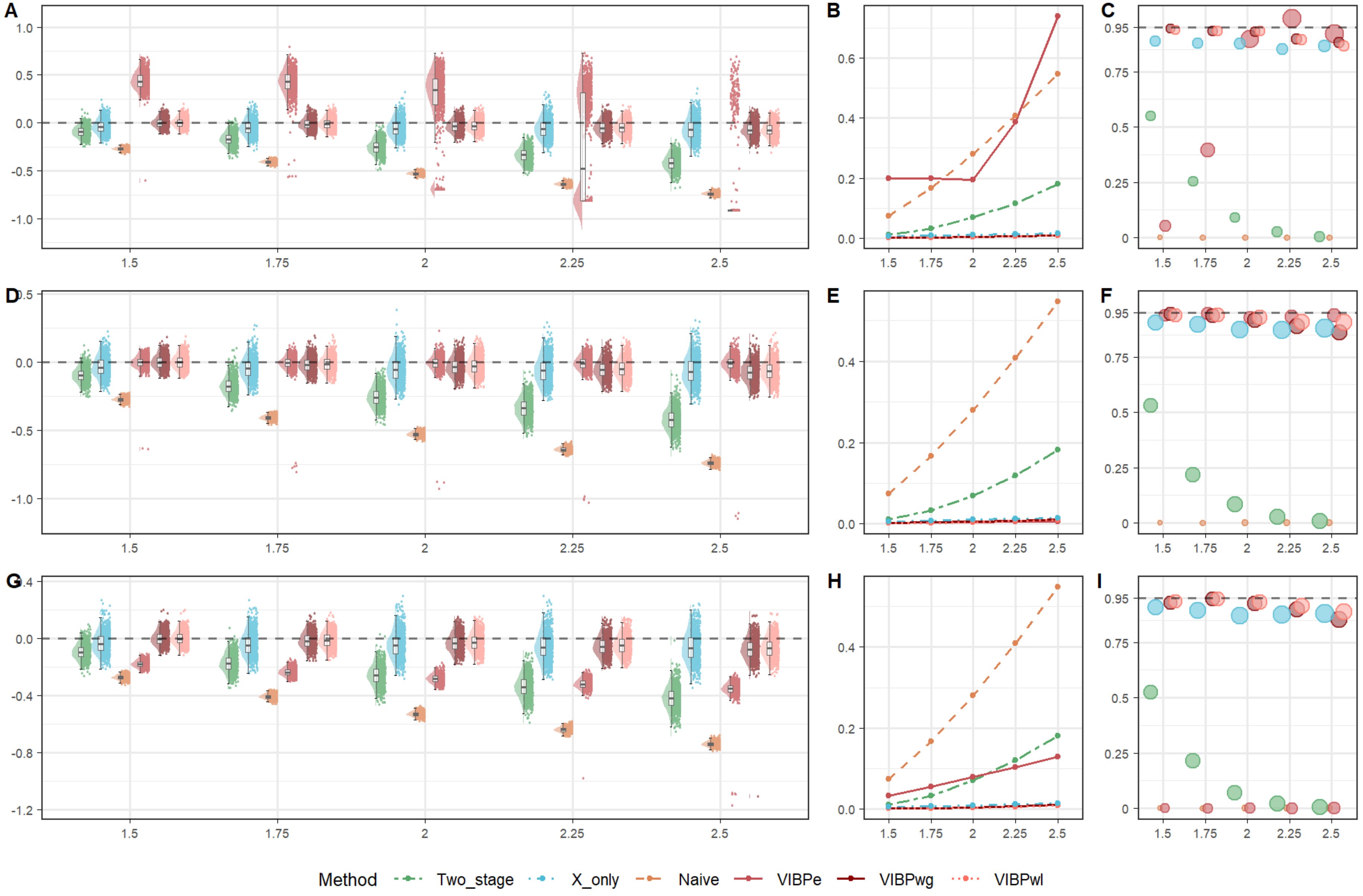

Figure S16. Comparison of operating characteristics under scenario 9 (Weibull high scale scenario) with censoring rate 0.1 for the VIBPe, VIBPwg, VIBPwl, naive, x-only, and two-stage methods. A. Bias under ρ=0.5; B. MSE under ρ=0.5; C. Coverage rate under ρ=0.5; D. Bias under ρ=1.0; E. MSE under ρ=1.0; F. Coverage rate under ρ=1.0; G. Bias under ρ=1.5; H. MSE under ρ=1.5; I. Coverage rate under ρ=1.5. The true effect is indexed on the HR scale ($HR = \exp(\beta)$) for presentation, whereas Bias, MSE, and coverage are computed for β on the log-HR scale.

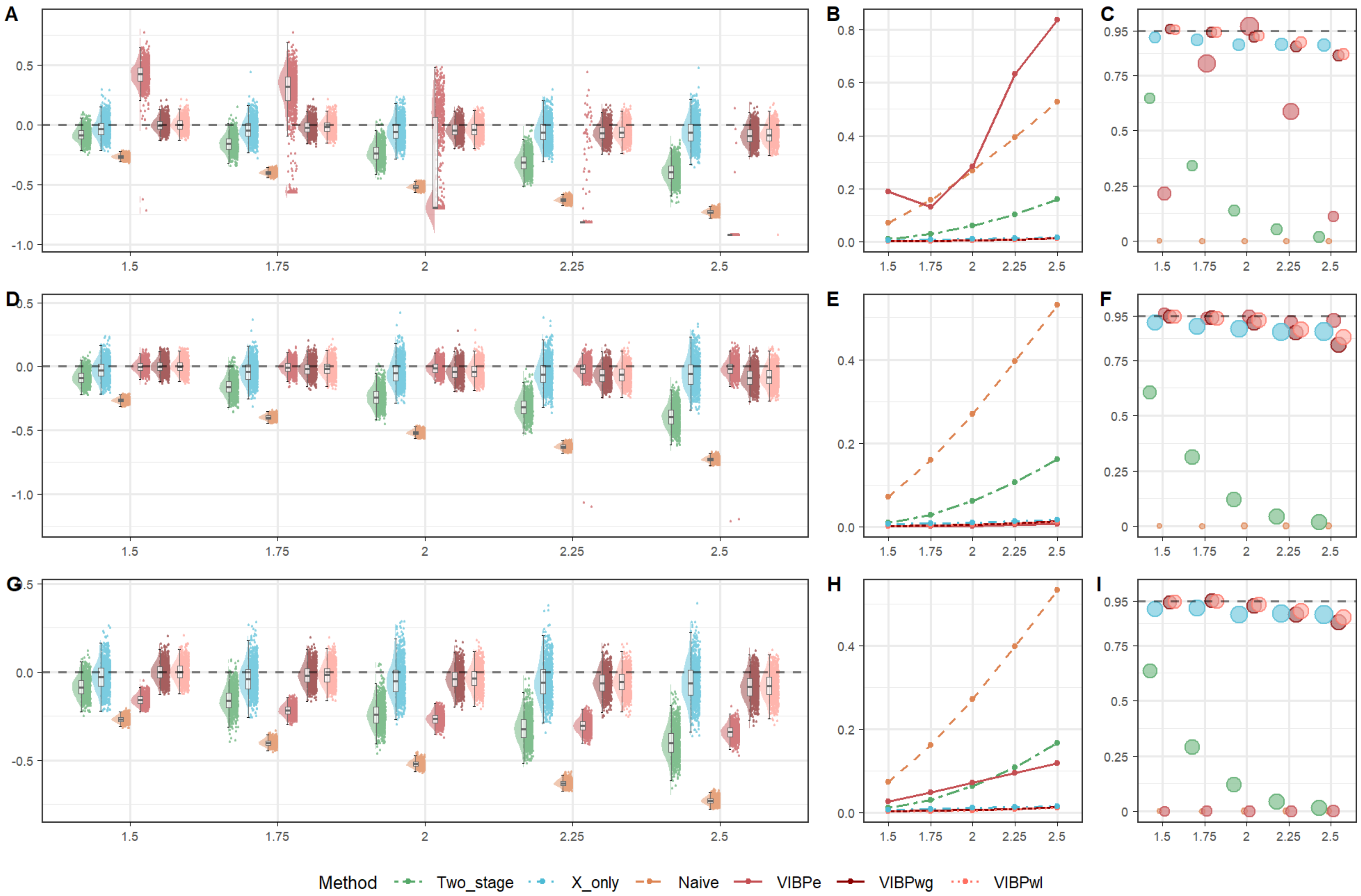

Figure S17. Comparison of operating characteristics under scenario 9 (Weibull high scale scenario) with censoring rate 0.3 for the VIBPe, VIBPwg, VIBPwl, naive, x-only, and two-stage methods. A. Bias under ρ=0.5; B. MSE under ρ=0.5; C. Coverage rate under ρ=0.5; D. Bias under ρ=1.0; E. MSE under ρ=1.0; F. Coverage rate under ρ=1.0; G. Bias under ρ=1.5; H. MSE under ρ=1.5; I. Coverage rate under ρ=1.5. The true effect is indexed on the HR scale ($HR = \exp(\beta)$) for presentation, whereas Bias, MSE, and coverage are computed for β on the log-HR scale.

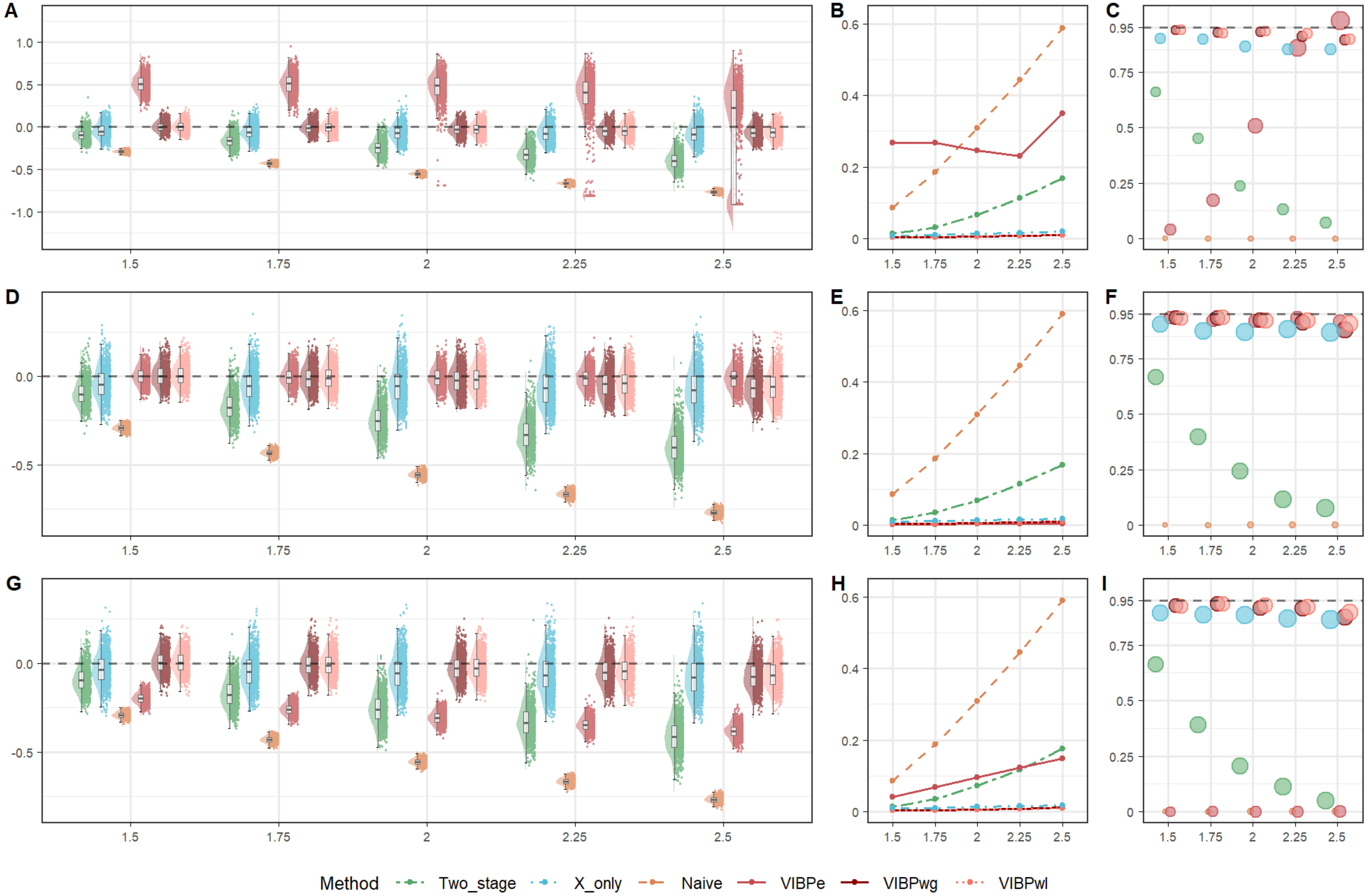

Figure S18. Comparison of operating characteristics under scenario 10 (Weibull high noise scenario) with censoring rate 0.1 for the VIBPe, VIBPwg, VIBPwl, naive, x-only, and two-stage methods. A. Bias under ρ=0.5; B. MSE under ρ=0.5; C. Coverage rate under ρ=0.5; D. Bias under ρ=1.0; E. MSE under ρ=1.0; F. Coverage rate under ρ=1.0; G. Bias under ρ=1.5; H. MSE under ρ=1.5; I. Coverage rate under ρ=1.5. The true effect is indexed on the HR scale ($HR = \exp(\beta)$) for presentation, whereas Bias, MSE, and coverage are computed for β on the log-HR scale.

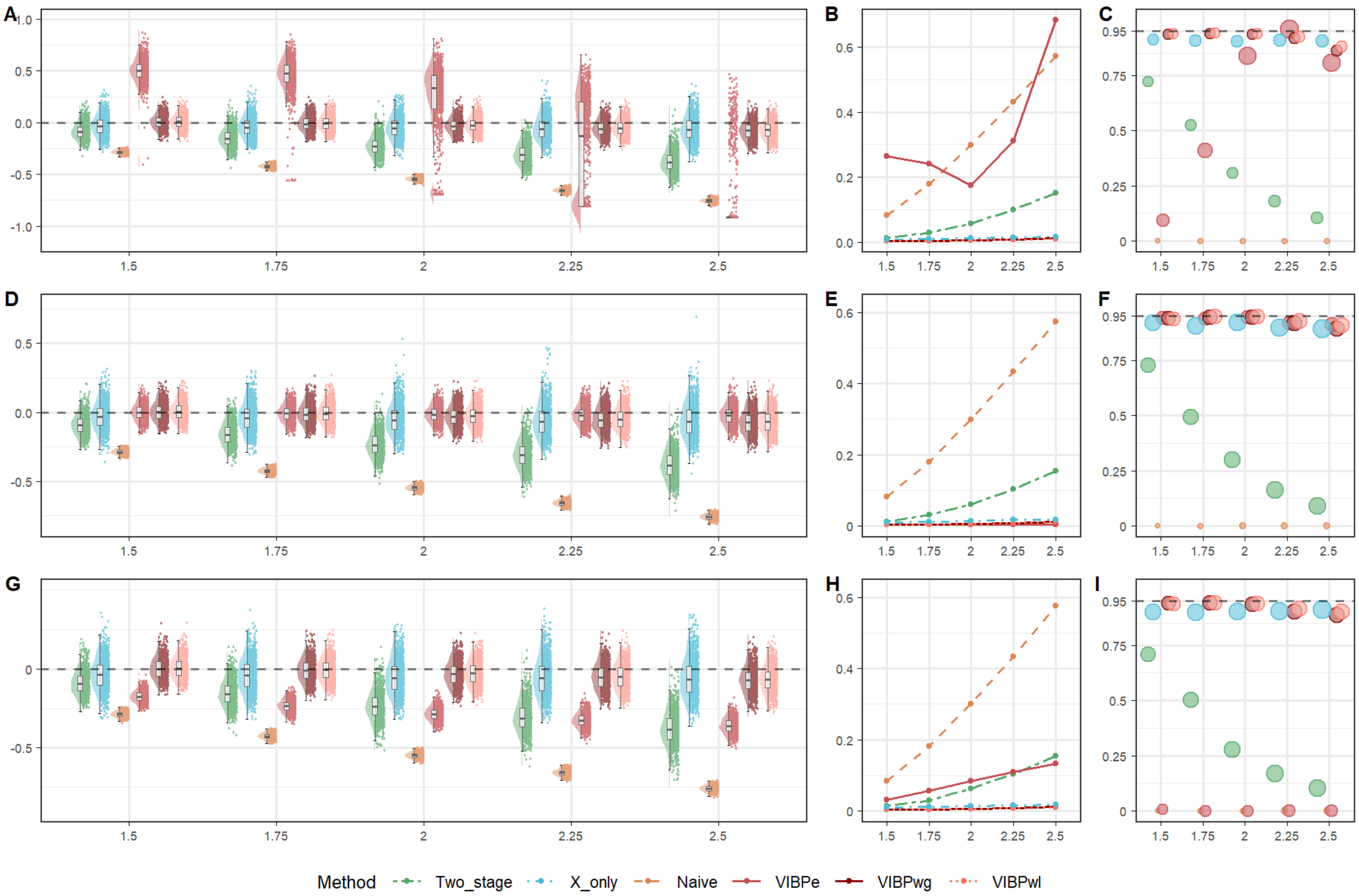

Figure S19. Comparison of operating characteristics under scenario 10 (Weibull high noise scenario) with censoring rate 0.3 for the VIBPe, VIBPwg, VIBPwl, naive, x-only, and two-stage methods. A. Bias under ρ=0.5; B. MSE under ρ=0.5; C. Coverage rate under ρ=0.5; D. Bias under ρ=1.0; E. MSE under ρ=1.0; F. Coverage rate under ρ=1.0; G. Bias under ρ=1.5; H. MSE under ρ=1.5; I. Coverage rate under ρ=1.5. The true effect is indexed on the HR scale ($HR = \exp(\beta)$) for presentation, whereas Bias, MSE, and coverage are computed for β on the log-HR scale.

S.E Schematic figure

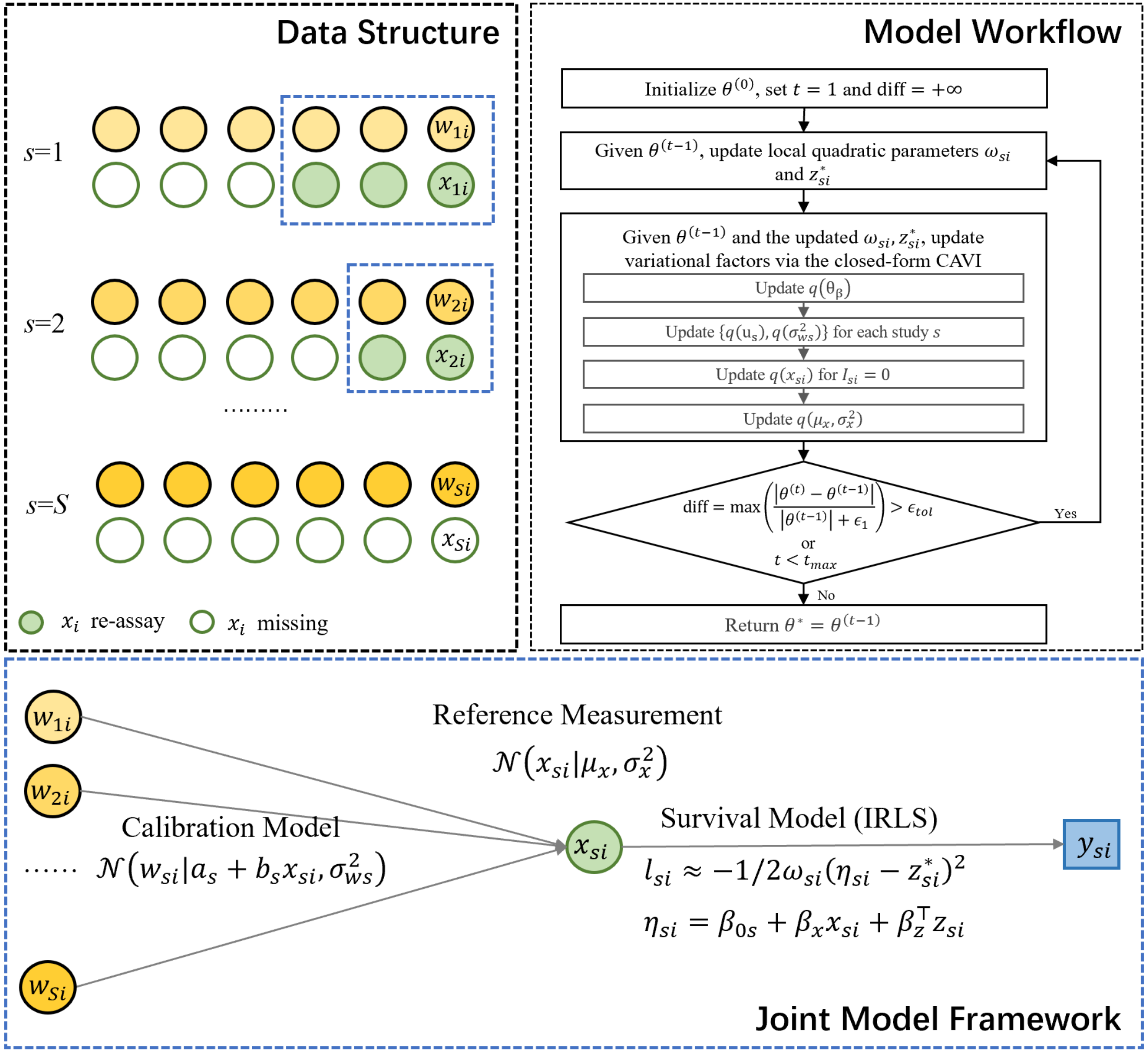

Figure S20. Schematic figure of the data structure, model workflow, and joint model framework.
